# Microbial Functional Gene Diversity Predicts Groundwater Contamination and Ecosystem Functioning

**DOI:** 10.1128/mBio.02435-17

**Published:** 2018-02-20

**Authors:** Zhili He, Ping Zhang, Linwei Wu, Andrea M. Rocha, Qichao Tu, Zhou Shi, Bo Wu, Yujia Qin, Jianjun Wang, Qingyun Yan, Daniel Curtis, Daliang Ning, Joy D. Van Nostrand, Liyou Wu, Yunfeng Yang, Dwayne A. Elias, David B. Watson, Michael W. W. Adams, Matthew W. Fields, Eric J. Alm, Terry C. Hazen, Paul D. Adams, Adam P. Arkin, Jizhong Zhou

**Affiliations:** a Environmental Microbiomics Research Center, School of Environmental Science and Engineering, Sun Yat-Sen University, Guangzhou, China; b Guangdong Provincial Key Laboratory of Environmental Pollution Control and Remediation Technology, School of Environmental Science and Engineering, Sun Yat-Sen University, Guangzhou, China; c Institute for Environmental Genomics, University of Oklahoma, Norman, Oklahoma, USA; d Department of Microbiology and Plant Biology, University of Oklahoma, Norman, Oklahoma, USA; e School of Civil Engineering and Environmental Sciences, University of Oklahoma, Norman, Oklahoma, USA; f School of Environment, Tsinghua University, Beijing, China; g Biosciences Division, Oak Ridge National Laboratory, Oak Ridge, Tennessee, USA; h Department of Civil and Environmental Engineering, University of Tennessee, Knoxville, Tennessee, USA; i Department of Biochemistry and Molecular Biology, University of Georgia, Athens, Georgia, USA; j Department of Microbiology and Immunology, Montana State University, Bozeman, Montana, USA; k Biological Engineering Department, Massachusetts Institute of Technology, Cambridge, Massachusetts, USA; l Earth and Environmental Sciences, Lawrence Berkeley National Laboratory, Berkeley, California, USA; m Department of Bioengineering, University of California, Berkeley, California, USA; University of California, Irvine

**Keywords:** groundwater microbiome, random forest, ecosystem functioning, environmental contamination, metagenomics, microbial functional gene

## Abstract

Contamination from anthropogenic activities has significantly impacted Earth’s biosphere. However, knowledge about how environmental contamination affects the biodiversity of groundwater microbiomes and ecosystem functioning remains very limited. Here, we used a comprehensive functional gene array to analyze groundwater microbiomes from 69 wells at the Oak Ridge Field Research Center (Oak Ridge, TN), representing a wide pH range and uranium, nitrate, and other contaminants. We hypothesized that the functional diversity of groundwater microbiomes would decrease as environmental contamination (e.g., uranium or nitrate) increased or at low or high pH, while some specific populations capable of utilizing or resistant to those contaminants would increase, and thus, such key microbial functional genes and/or populations could be used to predict groundwater contamination and ecosystem functioning. Our results indicated that functional richness/diversity decreased as uranium (but not nitrate) increased in groundwater. In addition, about 5.9% of specific key functional populations targeted by a comprehensive functional gene array (GeoChip 5) increased significantly (*P* < 0.05) as uranium or nitrate increased, and their changes could be used to successfully predict uranium and nitrate contamination and ecosystem functioning. This study indicates great potential for using microbial functional genes to predict environmental contamination and ecosystem functioning.

## IMPORTANCE

Disentangling the relationships between biodiversity and ecosystem functioning is an important but poorly understood topic in ecology. Predicting ecosystem functioning on the basis of biodiversity is even more difficult, particularly with microbial biomarkers. As an exploratory effort, this study used key microbial functional genes as biomarkers to provide predictive understanding of environmental contamination and ecosystem functioning. The results indicated that the overall functional gene richness/diversity decreased as uranium increased in groundwater, while specific key microbial guilds increased significantly as uranium or nitrate increased. These key microbial functional genes could be used to successfully predict environmental contamination and ecosystem functioning. This study represents a significant advance in using functional gene markers to predict the spatial distribution of environmental contaminants and ecosystem functioning toward predictive microbial ecology, which is an ultimate goal of microbial ecology.

## INTRODUCTION

Anthropogenic activities have impacted Earth’s biosphere through climate change, contamination of air, water, and soil environments, introduction of invasive species, depletion of natural resources, and alterations of biogeochemical cycling (1, 2). These activities have reduced biodiversity, destabilized ecosystem functions such as carbon (C) and nitrogen (N) cycles, and threatened human health (3–7). A recent study showed that several distinct factors, such as concentrations of sulfate, iron, and dissolved CH_4_ and H_2_, might control the composition of groundwater microbiomes and that the microbial functional diversity (FD) could explain groundwater chemistry in a pristine aquifer (8). However, the ecological consequences and mechanisms of environmental contamination in the biodiversity of microbial communities and ecosystem functioning remain largely unclear. Even more challenging is to establish linkages between microbial biodiversity and ecosystem functioning.

It is generally believed that FD is better than taxonomic diversity (TD) and/or phylogenetic diversity (PD) for predicting ecosystem functioning (9–12). For example, a recent study across a gradient of sites from the subarctic to the tropics showed that a reduction of decomposer FD consistently decreased the rate of litter decomposition and carbon and nitrogen cycling (13). However, how to select molecular functional predictors (e.g., functional genes) remains a challenging question (11). Functional gene arrays (e.g., GeoChip) target key genes involved in geochemical cycles, bioremediation, stress responses, and other environmental processes and have been widely used to functionally profile microbial communities (14–19). Therefore, GeoChip is an ideal tool to examine the impacts of environmental contaminants on groundwater microbiomes.

The Oak Ridge Integrated Field Research Challenge (OR-IFRC) experimental site, located in Bear Creek Valley, Oak Ridge, TN, is a legacy site for the early development of enriched uranium (U) under the Manhattan Project. At this site, numerous studies have been conducted to examine the impact of contaminants on biological communities and ecosystem functioning (20–29). For example, a metagenome analysis of FW106, a highly contaminated well, showed that high relative levels of abundance of key genes encoding geochemical resistance functions were required for microbial survival in the presence of known environmental contaminants at the site (20). Also, key functional groups have been isolated and identified from the OR-IFRC site, including sulfate-reducing bacteria (SRB), nitrate-reducing bacteria (NRB), and metal-reducing bacteria (MRB) like *Anaeromyxobacter*, *Clostridium*, *Desulfovibrio*, *Desulfitobacterium*, *Geobacter*, *Hyphomicrobium*, *Intrasporangium*, *Pseudomonas*, and *Rhodanobacter* species (20, 23, 25–29). Recently, groundwater from 93 noncontaminated and contaminated wells along the Bear Creek Valley at the OR-IFRC site were sampled. Those wells had a wide range of environmental gradients and associated ecosystem data (22), thus making it possible to use microbial community data for predicting groundwater contamination. The results showed that 16S rRNA gene-sequencing analysis of groundwater microbiomes could accurately identify environmental contaminants (e.g., uranium or nitrate) at the OR-IFRC site (22). However, taxonomic information alone may not be enough to reflect the functional aspects of microbial communities or ecosystems, as not all members of a taxon may carry certain functional genes, making it difficult to predict geochemical properties, especially ecosystem functioning. The following important questions remain to be addressed. (i) How does the functional diversity of groundwater microbiomes change across a range of environmental gradients (e.g., pH, uranium, and nitrate)? (ii) What specific functional genes/populations are stimulated under high concentrations of uranium and nitrate? (iii) Is it possible to predict environmental contamination (e.g., uranium or nitrate) and ecosystem functioning using microbial functional genes?

In this study, we hypothesized the following: (i) FD would decrease with increased environmental contamination (e.g., uranium or nitrate) or a significant change in environmental conditions (e.g., pH); (ii) under conditions of uranium and nitrate contamination, the abundance of some key functional genes/populations (e.g., *dsrA* and cytochrome genes for uranium reduction or *nirK* and *napA* for nitrate reduction) would increase, while the rest would decrease or remain unchanged; and (iii) the relationship between FD and environmental contamination or ecosystem functioning would be predictable based on key microbial functional genes. To test those hypotheses, we used a new version of a functional-gene microarray (GeoChip 5.0) to analyze groundwater microbiomes from 69 wells at the OR-IFRC site. GeoChip is able to quantitatively detect known microbial functions but generally does not target unknown functions from known or unknown microbial groups. Our results indicate that the overall FD decreased as uranium (but not nitrate) concentrations increased or at low or high pH; however, some specific functional genes/populations were stimulated in response to uranium and nitrate contamination. Such microbial functional genes could be used to successfully predict uranium and nitrate contamination and ecosystem functioning. This study provides new insights for our understanding of the impacts of environmental contaminants on groundwater microbiomes and demonstrates the predictive power of microbial functional genes for environmental contamination and ecosystem functioning.

## RESULTS

### Geochemical properties and ecosystem function indicators.

A total of 38 environmental variables were measured, including pH, contaminant (e.g., uranium and nitrate) concentrations, dissolved gases (e.g., CO_2_, CH_4_, N_2_O, and H_2_S) as ecosystem function indicators, dissolved C and N, and direct cell counts, which were largely used in this study (see Table S1 in the supplemental material). The 69 wells had wide ranges of uranium, nitrate, and pH levels, with uranium at 0 to 55.3 mg/liter (average, 1.5 mg/liter), nitrate at 0 to 11,648 mg NO_3_^−^-N (nitrate as nitrogen)/liter (average, 641 mg NO_3_^−^-N/liter), and pHs of 3 to 10.5 (average, pH 6.9). Furthermore, wells with high concentrations of uranium (e.g., >3 mg/liter) also had high concentrations of nitrate (1,516 to 11,648 mg NO_3_^−^-N/liter) and low pHs (3.0 to 5.2). Also, dissolved gases varied greatly, with CO_2_ at 0 to 29,739 mg/liter (average, 476 mg/liter), N_2_O at 0 to 1.2 mg/liter (average, 0.1 mg/liter), CH_4_ at 0 to 0.6 mg/liter (average, close to 0 mg/liter), and H_2_S at 0 to 4.2 mg/liter (average, 0.1 mg/liter). The amounts of bacterial biomass in groundwater samples ranged from 3.5 × 10^2^ to 1.8 × 10^6^ cells/ml (average, 1.2 × 10^5^ cells/ml), while the levels of dissolved organic carbon (DOC) were 0.2 to 128.2 mg/liter (average, 7.8 mg/liter) and the levels of dissolved inorganic carbon (DIC) were 9.4 to 179.2 mg/liter (average, 58.3 mg/liter). Such large ranges of environmental gradients provide an advantage in testing the relationships between functional gene diversity and environmental contamination, as well as ecosystem functioning.

10.1128/mBio.02435-17.3 TABLE S1  Key geochemical and ecosystem data for the 69 wells selected. Download TABLE S1, DOCX file, 0.03 MB. Copyright © 2018 He et al. 2018 He et al. This content is distributed under the terms of the Creative Commons Attribution 4.0 International license.

### The relationships between functional richness/diversity/abundance, microbial biomass, and contaminant concentrations.

**FIG 1:**
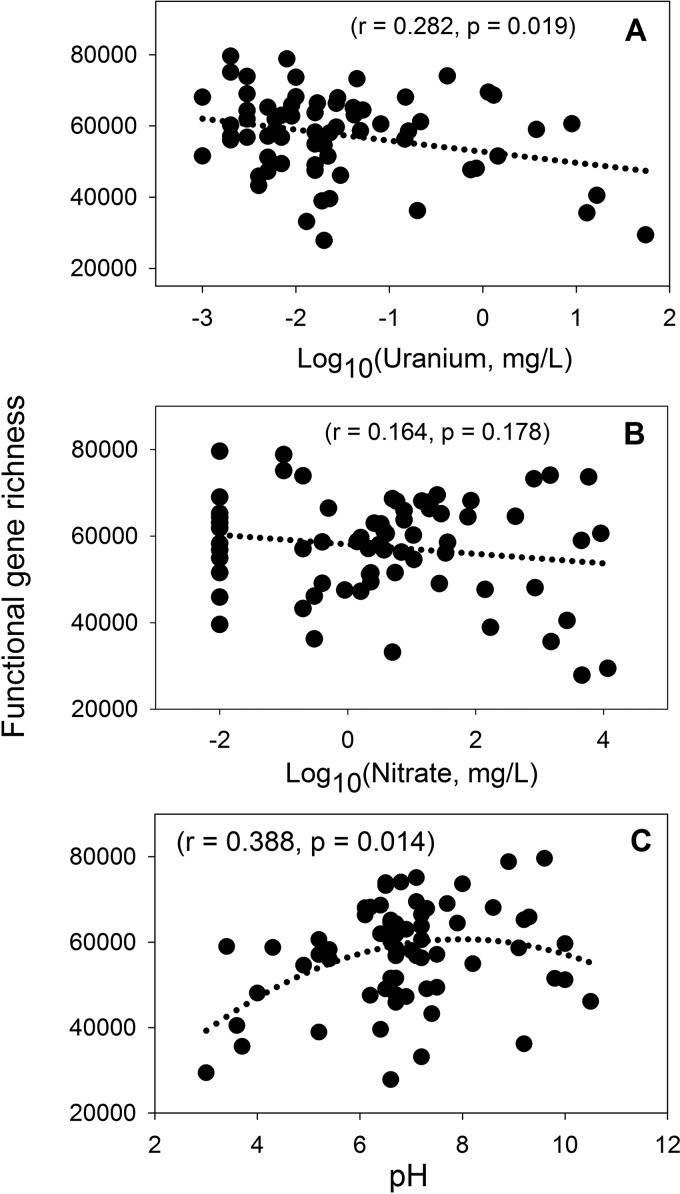
Relationships between the overall functional richness and concentrations of uranium (A) and nitrate (B), as well as pH (C), in groundwater. Uranium and nitrate concentrations were first log transformed, and then linear regressions were performed for functional richness and uranium or nitrate concentrations. Nonlinear regression was used for functional richness and pH.

**FIG 2:**
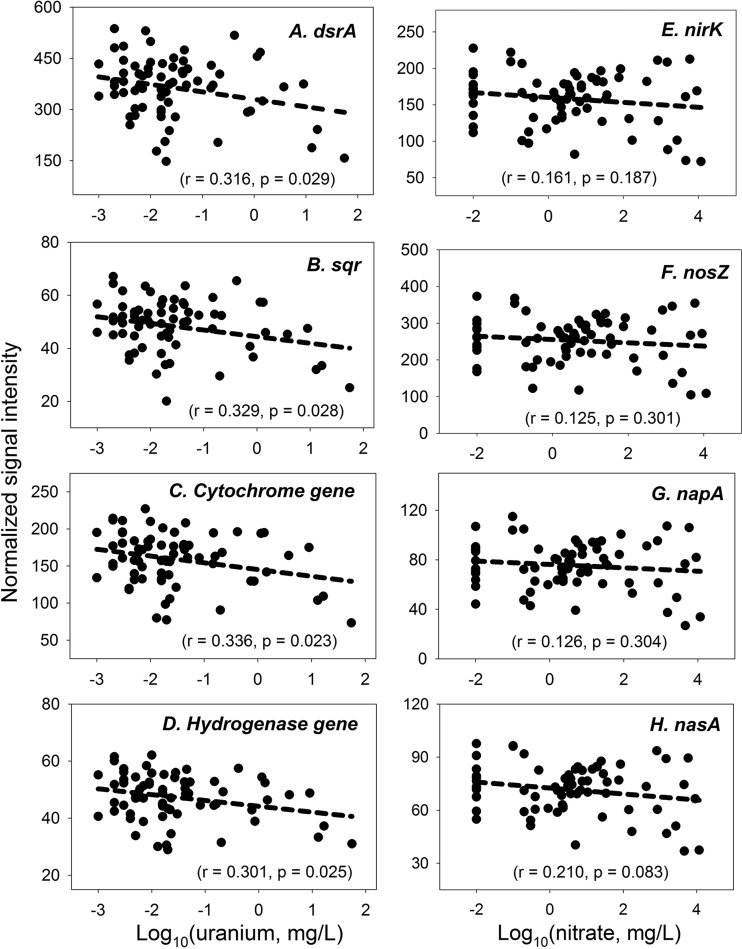
Linear relationships between the levels of abundance of specific functional gene families and log-transformed Uranium (A to D) or nitrate (E to H) concentrations in groundwater, including data for *dsrA*, encoding the alpha subunit of sulfite reductase for dissimilatory sulfite reduction (A), *sqr*, encoding sulfide-quinone reductase (B), cytochrome genes from well-known organisms, e.g., *Geobacter*, *Anaeromyxobacter*, *Dechloromonas*, *Desulfovibrio*, *Shewanella*, *Desulfurobacterium*, *Desulfobacterium*, *Rhodobacter*, *Pseudomonas*, *Enterobacter*, and *Ochrobactrum* (C), hydrogenase genes from well-known organisms, e.g., *Geobacter*, *Desulfovibrio*, *Desulfurobacterium*, *Desulfobacterium*, and *Rhodobacter* (D), *nirK*, encoding nitrite reductase for denitrification (E), *nosZ*, encoding nitrous oxide reductase for denitrification (F), *napA*, encoding nitrate reductase for dissimilatory nitrate reduction (G), and *nasA*, encoding nitrate reductase for assimilatory nitrate reduction (H).

For this study, we defined functional richness as the number of functional genes detected by GeoChip 5.0 and functional diversity as the Shannon diversity index. The overall levels of functional gene richness and diversity decreased significantly (*P* < 0.05) as uranium concentrations increased. The functional diversity was highest at a neutral pH, not under low- or high-pH conditions, but it was not significantly (*P* < 0.05) impacted by nitrate concentrations in groundwater (Fig. 1; Fig. S1). We further examined the relationships between the levels of abundance of key gene families along the environmental gradients. For example, the abundances of sulfur (S) cycling genes (e.g., *dsrA* and *sqr*) and cytochrome and hydrogenase genes decreased significantly (*P* < 0.05) with increasing uranium concentrations (Fig. 2A to D). However, the abundances of denitrification (e.g., *nirK* and *nosZ*), dissimilatory N reduction (e.g., *napA*), and assimilatory N reduction (e.g., *nasA*) genes did not decrease significantly (*r* = 0.125 to 0.210, *P* > 0.05) with increasing nitrate concentrations (Fig. 2E to H). Further analysis of other key N cycling genes showed significantly (*P* < 0.05) decreased abundances with increased uranium or at low or high pHs, but no significant (*P* > 0.05) correlations were observed between N cycling gene abundances and nitrate concentrations (Table S2). In addition, the effects of uranium and pH on microbial biomass (measured by direct cell count) were not significant (*P* > 0.05), nor was there a significant correlation (*P* > 0.05) between biomass and functional richness, but it appeared that microbial biomass increased significantly (*P* = 0.001) with increased nitrate concentrations, suggesting that nitrate consumers (e.g., nitrate reducers) may be dominant in the environment (Fig. S2). Further analysis showed that the abundance of ~95% of genes detected by GeoChip 5.0 decreased, while only about 5% of them increased, indicating that most of the functional genes were inhibited or remained unchanged as uranium and nitrate concentrations increased.

10.1128/mBio.02435-17.1 FIG S1  Relationships between the overall functional diversity (Shannon diversity index) and concentrations (log transformed) of uranium (A) and nitrate (B), as well as pH (C), in groundwater. Linear regression was used for the Shannon index and uranium or nitrate concentrations, and nonlinear regression for the Shannon index and pHs. Download FIG S1, TIF file, 0.7 MB. Copyright © 2018 He et al. 2018 He et al. This content is distributed under the terms of the Creative Commons Attribution 4.0 International license.

10.1128/mBio.02435-17.2 FIG S2  Relationships between log-transformed uranium (A) or nitrate (B) concentrations, pH (C), or functional richness (D) and groundwater microbial biomass determined by the acridine orange direct count (AODC) method. Linear regression was used for such relationships. Download FIG S2, TIF file, 0.5 MB. Copyright © 2018 He et al. 2018 He et al. This content is distributed under the terms of the Creative Commons Attribution 4.0 International license.

10.1128/mBio.02435-17.4 TABLE S2  Regressions of key nitrogen cycling gene abundance with uranium, nitrate, or pH. Uranium and nitrate concentrations were first log transformed and then used for linear regressions, while nonlinear regression without log transformation was used for pHs. *P* values of <0.05 are in boldface. Download TABLE S2, DOCX file, 0.01 MB. Copyright © 2018 He et al. 2018 He et al. This content is distributed under the terms of the Creative Commons Attribution 4.0 International license.

### Key functional populations stimulated in response to a uranium gradient.

**FIG 3:**
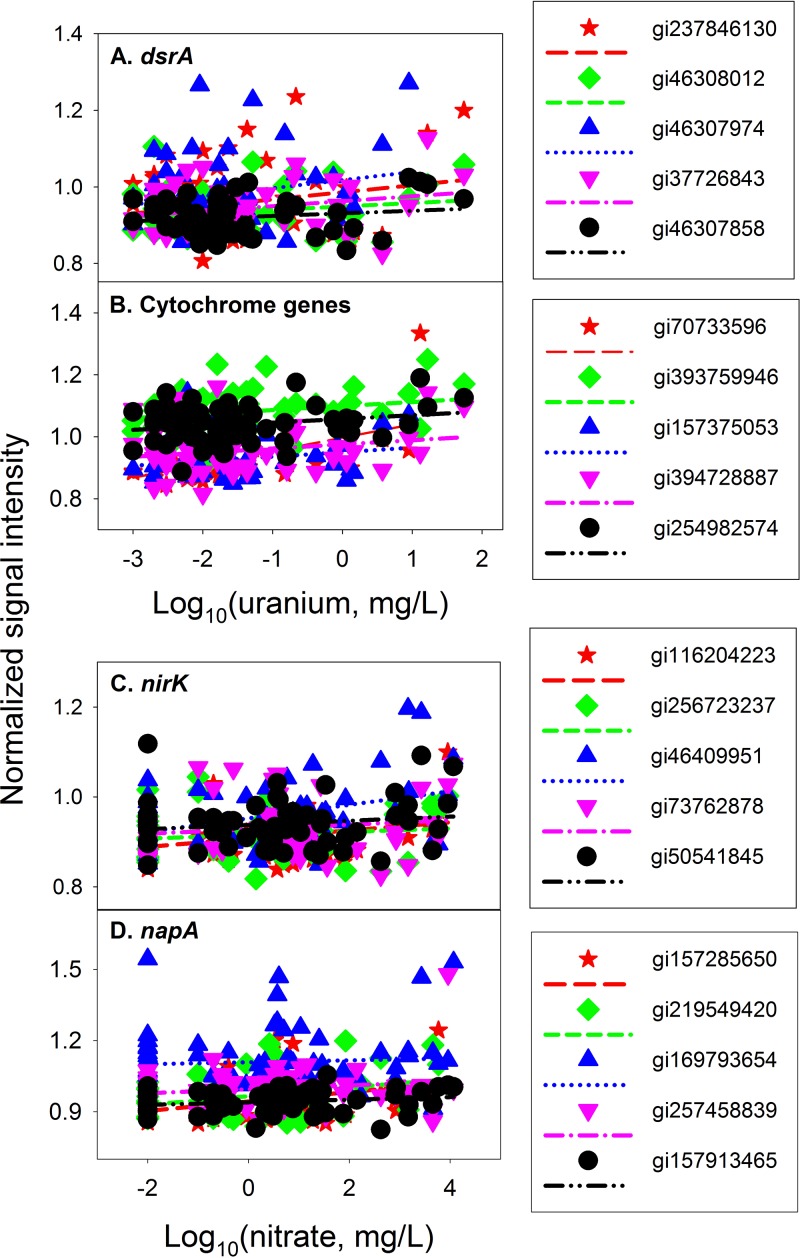
Significantly (*P* < 0.05) positive correlations between the levels of abundance of stimulated populations and log-transformed uranium (A and B) or nitrate (C and D) concentrations, including data for *dsrA* gene variants gi237846130, gi46308012, gi46307974, gi37726843, and gi46307858, derived from uncultured sulfate-reducing bacteria (A), cytochrome genes gi70733596 from *Pseudomonas fluorescens*, gi393759946 from *Alcaligenes faecalis*, gi157375053 from *Shewanella sediminis*, gi394728887 from *Enterobacter* sp., and gi254982574 from *Geobacter* sp. (B), *nirK* gene variants gi116204223 from *Chaetomium globosum*, gi256723237 from *Nectria haematococca*, and gi46409951, gi73762878, and gi50541845 from uncultured denitrifying bacteria (C), and *napA* gene variants gi219549420 from *Vibrio parahaemolyticus*, gi257458839 from *Campylobacter gracilis*, gi157913465 from *Dinoroseobacter shibae*, and gi157285650 and gi169793654 from uncultured nitrate-reducing bacteria (D).

Although the richness and diversity of functional genes generally decreased as uranium concentrations increased in groundwater, some specific populations of certain functional gliders did increase significantly (*P* < 0.05) (Fig. 3A and B; Table S3). For example, the abundance of 43 *dsrA*-bearing populations (~5.8% of total *dsrA* detected by GeoChip 5), mostly uncultured SRB with a few sequenced species (e.g., *Halorhodospira halophila*, *Desulfobulbus propionicus*, *Pelodictyon luteolum*, and *Vibrio rotiferianus*), increased significantly (*P* < 0.05) (Table S3). In particular, five abundant *dsrA* probes/gene variants (gi237846130, gi46308012, gi46307974, gi37726843, and gi46307858) derived from uncultured SRB were identified as being significantly (*P* < 0.05) increased as uranium increased (Fig. 3A). Increased levels of abundance of 21 cytochrome (~4.6%) and 6 hydrogenase (~7.3%) gene variants were also observed, specifically from well-known microorganisms like *Geobacter*, *Dechloromonas*, *Enterobacter*, *Pseudomonas*, *Alcaligenes*, *Desulfovibrio*, *Desulfitobacterium*, *Rhodobacter*, *Ochrobactrum*, and *Anaeromyxobacter* (Table S3). Also, five abundant cytochrome genes (gi70733596, gi393759946, gi157375053, gi394728887, and gi254982574) were significantly (*P* < 0.05) increased as uranium concentrations increased in groundwater (Fig. 3B). These stimulated populations could play important roles in uranium bioremediation at this site.

10.1128/mBio.02435-17.5 TABLE S3  Relationships between the abundances of significantly increased or decreased populations (bearing key genes) and uranium concentrations by linear regression. Significantly increased slopes are in boldface, and the relative abundances are presented as mean ratios. Download TABLE S3, DOCX file, 0.03 MB. Copyright © 2018 He et al. 2018 He et al. This content is distributed under the terms of the Creative Commons Attribution 4.0 International license.

### Key functional populations stimulated in response to a nitrate gradient.

We also found that the abundance of many specific functional genes/populations involved in N cycling increased significantly (*P* < 0.05) as nitrate increased (Fig. 3C and D; Table S4). For example, the abundance of 13 *nirK*-bearing (4.9%) populations increased significantly (*P* < 0.05), with most being uncultured bacteria and a few sequenced microbes (e.g., *Chaetomium*, *Arthroderma*, *Nectria*, and *Pseudomonas*); the abundance of 9 *napA* (6.0%) gene variants for dissimilatory N reduction, derived from *Beggiatoa*, *Vibrio*, *Campylobacter*, and *Dinoroseobacter* species, as well as uncultured NRB, also increased significantly (*P* < 0.05) as nitrate increased (Table S4). Five abundant *nirK* gene variants (gi116204223, gi256723237, gi46409951, gi73762878, and gi50541845) (Fig. 3C) and five abundant *napA* gene variants (gi157285650, gi219549420, gi169793654, gi257458839, and gi157913465) increased significantly (*P* < 0.05) as nitrate increased (Fig. 3D). In addition, populations stimulated by high concentrations of nitrate were observed for other N cycling genes, such as *amoA*, *nifH*, *narG*, *nirS*, *norB*, *nasA*, *nosZ*, and *nrfA* (Table S4). These stimulated populations are expected to play important roles in bioremediation of this nitrate-contaminated site.

10.1128/mBio.02435-17.6 TABLE S4  Relationships between the abundances of significantly increased or decreased populations (bearing key genes) and nitrate concentrations by linear regression. Significantly increased slopes are in boldface, and the relative abundances are presented as mean ratios. Download TABLE S4, DOCX file, 0.1 MB. Copyright © 2018 He et al. 2018 He et al. This content is distributed under the terms of the Creative Commons Attribution 4.0 International license.

### Prediction of uranium contamination in groundwater using microbial functional genes.

As significant relationships were observed between functional richness, diversity, and/or populations and uranium concentrations in groundwater, we attempted to predict groundwater contamination by the presence of microbial functional genes using random forest, a machine learning method (30). First, we selected a total of 2,361 of the functional genes detected that could predict uranium contamination on the basis of being involved in S cycling and electron transfer (e.g., *dsrA*, *dsrB*, *sir*, cytochrome, hydrogenase, and cytochrome P-450 genes). Cross-validation by out-of-bagging (OOB) estimation of errors for classification of uranium contamination was 28.99%. Second, we selected a subset of 1,521 specific functional genes from the first set of 2,361 genes for predicting uranium contamination, including 892 *dsrA*, 536 cytochrome, and 93 hydrogenase genes. OOB estimation of errors was 24.64% for all three functional gene families and 24.64%, 26.09%, and 28.99% for *dsrA*, cytochrome, and hydrogenase genes, respectively, indicating that the best predictor for uranium contamination was *dsrA* or a combination of all three gene families, each with an error rate of 24.64%. Third, we used the significantly changed populations bearing the best predictor, *dsrA* (Table S3), and the same results were observed for uranium contamination prediction (Table 1). To further improve our prediction, we used the area under the receiver operating characteristic curve as the predictive accuracy for random forest (AUC-RF) (31) to automatically select 50 predictors (Table S5) from the initial 2,361 functional probes related to uranium reduction, which dramatically decreased the OOB estimate of error rate, from 28.99% to 11.59% (Table 1). These results indicated that microbial functional genes were able to successfully predict groundwater uranium contamination.

10.1128/mBio.02435-17.7 TABLE S5  Fifty predictors from 2,361 detected functional genes related to uranium reduction automatically selected by AUS-RF for predicting uranium contamination in groundwater. Items in boldface were also identified as belonging to populations significantly increased/decreased with increasing uranium concentrations in groundwater (see Table S3). Download TABLE S5, DOCX file, 0.02 MB. Copyright © 2018 He et al. 2018 He et al. This content is distributed under the terms of the Creative Commons Attribution 4.0 International license.

**TABLE 1:** Performance of the random forest model for predicting environmental contamination by uranium or nitrate in 69 wells at the OR-IFRC site using microbial functional genes as predictors

Contaminant	Predictor^a^	OOB error rate (%)	No. of wells predicted/no. of wells defined	
			Background wells^b^	Contaminated wells^c^
Uranium	All S cycling and metal-related genes	28.99	47/47	2/22
	All *dsrA*, cytochrome, and hydrogenase genes	24.64	47/47	5/22
	All *dsrA* genes	24.64	47/47	5/22
	All cytochrome genes	26.09	46/47	5/22
	All hydrogenase genes	28.99	41/47	8/22
	Key *dsrA*, cytochrome, and hydrogenase genes	27.54	45/47	5/22
	Key *dsrA* genes	24.64	45/47	7/22
	Key cytochrome genes	39.13	38/47	4/22
	Key hydrogenase genes	42.03	33/47	7/22
	AUC-RF selection	11.59	47/47	14/22

Nitrate	All N cycling genes	36.23	39/44	5/25
	All *nifH*, *amoA*, *narG*, *nasA*, and *napA* genes	34.78	40/44	5/25
	All *nifH* genes	33.33	41/44	5/25
	All *amoA* genes	27.54	41/44	9/25
	All *narG* genes	36.23	40/44	4/25
	All *nasA* genes	36.23	37/44	7/25
	All *napA* genes	34.78	41/44	4/25
	Key *nifH*, *amoA*, *narG*, *nasA*, and *napA* genes	30.43	40/44	8/25
	Key *nifH* genes	27.54	41/44	9/25
	Key *amoA* genes	28.99	39/44	10/25
	Key *narG* genes	37.68	37/44	6/25
	Key *nasA* genes	40.58	32/44	9/25
	Key *napA* genes	40.58	32/44	9/25
	AUC-RF selection	15.94	42/44	16/25

a Key functional genes detected from each family are listed in Tables S3 and S4 in the supplemental material.

b In background wells, the concentrations of uranium or nitrate were 30 µg/liter or below or 10 mg/liter or below, respectively.

c In contaminated wells, the concentrations of uranium or nitrate were higher than 30 µg/liter or 10 mg/liter, respectively.

### Prediction of nitrate contamination in groundwater using microbial functional genes.

Similarly, we predicted nitrate contamination in groundwater. First, we selected a total of 5,273 functional genes involved in N cycling and showed that the error rate for nitrate contamination prediction was 36.23%. Second, we selected a subset of 2,239 specific functional genes from that first set that were involved in N fixation (1,044 *nifH* genes), nitrification (173 *amoA* genes), denitrification (705 *narG* genes), and assimilatory (134 *nasA* genes) and dissimilatory (183 *napA* genes) N reduction, and the error rates were 34.79% for all the gene families selected and 33.33%, 27.54%, 36.23%, 36.23%, and 34.78%, respectively, for individual functional gene families, indicating that the best predictor for nitrate contamination was *amoA*, with an error rate of 27.54%. Third, we used the best predictor, *amoA*, and the significantly changed populations bearing it for the same prediction, and the error rate for nitrate contamination prediction was 28.99% (Table 1), which was not an improvement from the previous test. To reduce the collinearity, we again used AUC-RF (31) to automatically select 54 predictors (Table S6) from the original 5,273 N cycling genes. This substantially improved our prediction, decreasing the OOB estimate of error rate to 15.94% (Table 1). These results indicated that microbial functional genes were able to accurately predict nitrate contamination in groundwater.

10.1128/mBio.02435-17.8 TABLE S6  Fifty-four predictors from 5,273 detected N cycling genes automatically selected by AUC-RF for predicting nitrate contamination in groundwater. Items in boldface were also identified as belonging to populations significantly increased/decreased with increasing nitrate concentrations in groundwater (see Table S4). Download TABLE S6, DOCX file, 0.02 MB. Copyright © 2018 He et al. 2018 He et al. This content is distributed under the terms of the Creative Commons Attribution 4.0 International license.

### Prediction of ecosystem functioning using microbial functional genes.

**FIG 4:**
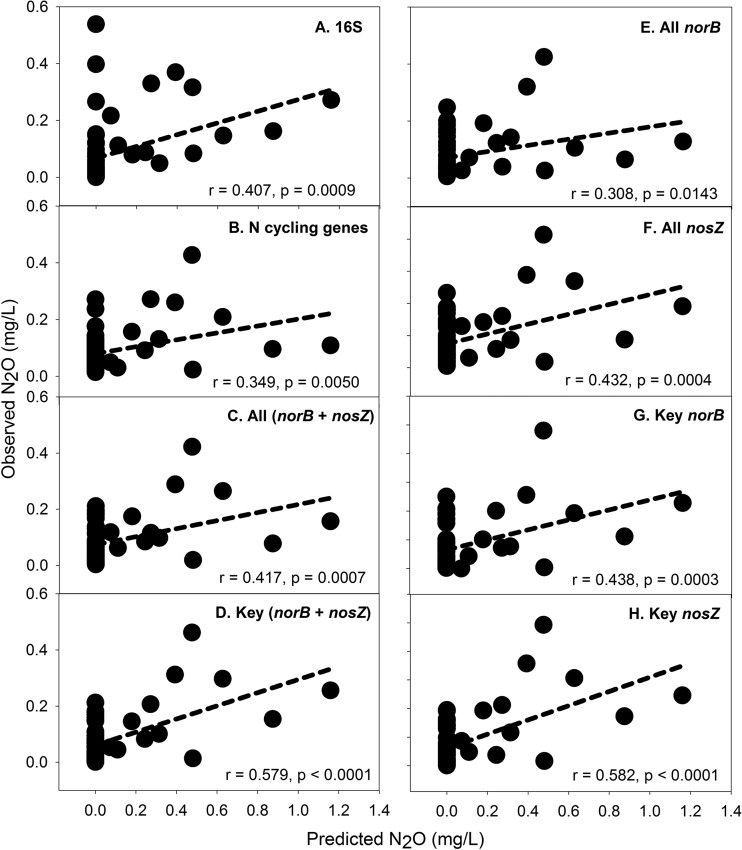
Random forest predictions of N_2_O concentrations in groundwater using different sets of genes, including 16S rRNA genes (A); all N cycling genes (B); all *norB* and *nosZ* genes (C); key (significantly increased/decreased) *norB* and *nosZ* genes (D); all *norB* genes (E); all *nosZ* genes (F); key *norB* genes (G); and key *nosZ* genes (H). All *norB* and *nosZ* key genes are listed in Table S4 in the supplemental material.

We also attempted to select specific microbial functional genes, as well as 16S rRNA genes (for a comparison), to predict ecosystem functions that may be occurring based on the concentrations of dissolved gases (e.g., CO_2_, CH_4_, and N_2_O) in the groundwater (Table S1). No significant correlations were observed either between the predicted CH_4_ concentration and the observed CH_4_ concentration or between the predicted CO_2_ concentration and the observed CO_2_ concentration (data not shown). However, when 16S rRNA genes, N cycling genes, all *norB* or *nosZ* genes, key *norB* or *nosZ* genes, all *norB* plus *nosZ* genes, or key *norB* plus *nosZ* genes were used to predict N_2_O concentrations in groundwater, significant correlations between the predicted N_2_O concentration and the observed N_2_O concentration were evident, and among those sets of genes or combinations of genes, key *norB* plus *nosZ* genes or key *nosZ* genes were the best predictors for N_2_O concentrations in groundwater based on the *r* and *P* values of linear regressions (Fig. 4). The results suggest that microbial functional genes are potentially useful and better than 16S rRNA genes for predicting ecosystem functions (e.g., N_2_O concentrations in groundwater).

## DISCUSSION

Understanding the impacts of contaminants on biological communities and predicting the effects of those communities on ecosystem functioning are important topics in ecology and environmental management. In this study, we surveyed the functional diversity and composition of groundwater microbial communities and their linkages with environmental contamination or ecosystem functioning at the OR-IFRC experimental site. Our results showed that the overall functional diversity/richness of groundwater microbiomes decreased as uranium (but not nitrate) concentrations increased or at low or high pHs. However, some specific functional genes/populations involved in uranium and/or nitrate reduction and denitrification were stimulated, and these functional genes could be used to predict environmental contamination (e.g., uranium or nitrate) and ecosystem functioning. In addition, unlike previous studies, which only had a limited number of samples/wells, this study analyzed 69 microbial communities from a large range of environmental gradients (e.g., uranium, nitrate, and pH), providing a more robust picture of the impact of human activities on biodiversity. The experimental results from this study generally support our hypotheses (with the exception of the relationship between nitrate and functional diversity).

Our first hypothesis was that the overall functional diversity/richness of groundwater microbiomes would decrease with an increase in environmental contamination (e.g., uranium or nitrate) or under extreme pH conditions. A previous clone library analysis of *nirS* and *nirK* genes from the same site found that novel *nirK* and *nirS* sequences were present in the contaminated groundwater and that the diversity of both gene families changed with contaminant (e.g., uranium or nitrate) concentrations (32). Also, a comparison of metagenomes from FW106 (a highly contaminated well) and FW301 (a background well) revealed that long-term exposure to low pHs and high concentrations of uranium, nitrate, and organic solvents resulted in decreased species diversity and loss of functional diversity (20, 24). Additionally, GeoChip analysis of a landfill leachate-contaminated aquifer showed that leachate from an unlined landfill impacted the diversity, composition, structure, and functional potential of groundwater microbiomes as a function of groundwater pH, DOC, and concentrations of sulfate and ammonia (33). In this study, we found that the overall functional diversity of groundwater microbial communities decreased under uranium contamination or extreme pH conditions, which is consistent with previous observations in groundwater (20, 32–36), as well as in the soil environment (37–40). Several possible mechanisms might be responsible for such a reduction in the functional diversity/richness. First, most microorganisms may not have developed efficient strategies for surviving/growing in such stressed environments, so their abundances would decrease to below detection level or even to extinction (20, 24). Second, if there are no appropriate mechanisms to deal with high uranium concentrations in the environment, uranium may accumulate in or be deposited on the cell surface, which could directly or indirectly inhibit specific key functional genes/enzymes, as well as associated pathways (41), resulting in a decrease in functional richness/diversity. Third, low pHs might reduce intracellular pH and disrupt the chemiosmotic gradient (42), impairing cellular metabolism. Fourth, high concentrations of uranium and nitrate and low pHs coexist in some wells (e.g., FW-021, FW-106, FW-126, and FW-410), which may cause additive impacts, further reducing the overall functional diversity/richness. These possibilities may lead to a decreased functional richness/diversity of groundwater microbial communities. However, the functional richness/diversity of certain specific gene families did not decrease significantly as nitrate concentrations increased. One possible explanation is that most microbes (e.g., nitrate reducers) might use nitrate or related N compounds (e.g., NO_2_^−^, NO, N_2_O, or NH_4_^+^) as electron donors/acceptors and sources of energy and assimilatory N, so that they were able to cope with such high nitrate concentrations. Indeed, a previous study indicated that elevated nitrate could stimulate microorganisms, especially those with diverse metabolic capabilities (43). Therefore, our results generally support the hypothesis that the overall functional richness/diversity of groundwater microbial communities decreases as uranium concentrations increase or under extreme pH conditions in groundwater.

Although the overall functional diversity/richness decreased as uranium concentrations increased or remained unchanged as nitrate concentrations increased, some key functional genes/populations involved in uranium or nitrate reduction/resistance would be expected to increase under high concentrations of uranium and nitrate. The *dsrA* gene, encoding the alpha subunit of dissimilatory sulfite reductase, an SRB biomarker indicating the ability to reduce sulfate and heavy metals (e.g., uranium) (44–47), and cytochrome genes (48, 49) were enriched. Previous studies also indicated that some of these functional genes/populations were stimulated under conditions of high concentrations of heavy metals (e.g., uranium and chromate) in this OR-IFRC site (50–53), the Uranium Mill Tailings Remedial Action site in Rifle, CO (54), and the chromate-contaminated Hanford site (55), suggesting the important role of these functions in metal (e.g., uranium and chromate) reduction. As nitrate is an important nutrient and electron acceptor for microorganisms, adequately high concentrations of nitrate in groundwater are expected to stimulate N cycling genes and associated processes. For example, a recent study indicated that elevated nitrate could enrich functional genes involved in C, N, S, and phosphorus (P) cycling, thus leading to the potential *in situ* bioremediation of polybrominated diphenyl ether (PBDE)- and polycyclic aromatic hydrocarbon (PAH)-contaminated sites (43). In the current study, we found that the abundances of about 5 to 6% *dsrA*, cytochrome, and N cycling genes were positively correlated with the uranium or nitrate concentrations. These genes were largely derived from SRB, NRB, and MRB, particularly those microorganisms with versatile metabolic capabilities (e.g., *Rhodanobacter*, *Geobacter*, *Pseudomonas*, *Alcaligenes*, *Desulfovibrio*, *Desulfitobacterium*, *Rhodobacter*, and *Anaeromyxobacter*). Some of these key microorganisms have been isolated from the OR-IFRC site (23, 25–29), and several key genes have been identified by shotgun metagenome sequencing (20, 24). The results generally support our second hypothesis, that key functional genes/populations involved in uranium reduction, nitrate reduction, and denitrification could be stimulated under high concentrations of uranium and nitrate. These significantly increased or decreased functional genes or populations were used to predict uranium and nitrate contamination and ecosystem functioning in this study, as they are expected to play important roles in this groundwater system.

Two recent studies compared different machine learning methods, one aimed at finding predictors of bacterial vaginosis (56) and the other at identifying environmental sensors in groundwater contamination (22), and both showed that random forest was a suitable approach for predictive analysis of microbial communities. Another study showed that 16S rRNA gene sequencing data of human fecal communities were good predictors of a city’s obesity level using random forest algorithms (57). Also, 16S rRNA gene sequencing of fecal samples was used to distinguish pediatric patients with inflammatory bowel disease (IBD) from patients with similar symptoms (58). At the OR-IFRC site, a recent study found that 16S rRNA gene sequencing data could be used to successfully predict most (26 out of 38) of the groundwater geochemical properties, such as uranium and nitrate concentrations and pHs (22). Although all these studies used 16S rRNA genes as predictors, it is believed that functional genes may be better predictors of ecosystem functions. Currently, some challenges remain in the use of functional genes as predictors. One challenge is to determine which functional genes or sets of functional genes are appropriate choices for given functions, phenotypes (e.g., disease), or processes (e.g., CO_2_ production), and another challenge is to accurately identify or measure a specific phenotype or functional process.

In this study, our results indicated that uranium and nitrate contamination were accurately predicted, specifically with AUC-RF (31), and we also successfully predicted dissolved N_2_O in groundwater. However, several challenges still remain in predicting other ecosystem functions, such as CO_2_ and CH_4_ concentrations in groundwater. First, only a few wells had relatively high concentrations of CH_4_ or CO_2_, while most wells had undetectable concentrations of these gases in the groundwater. Such a skewed distribution of data may affect our prediction accuracy. Second, the high diversity of functional genes/populations may present multiple instances of collinearity in the community, thus compromising our predictions. Indeed, when we used AUC-RF to reduce collinearity, the prediction error rates decreased dramatically, from approximately 29% to 12% for uranium contamination and from 36% to 16% for nitrate contamination. Third, it is hard to identify the specific functional genes responsible for some general functional processes. For example, groundwater CO_2_ could be generated from many C decomposition pathways and other physical or chemical pathways or consumed by autotrophy and chemical reactions, making it difficult to select specific genes for predicting this functional process and, thus, limiting the predictive power. Fourth, the relationship between dissolved gases and functional gene abundance may be subtle. The concentrations of gases in groundwater may not accurately reflect ecosystem functioning, or functional gene abundance may not reflect actual activity. Perhaps due to these challenges, a recent study also showed that adding functional information did not improve classification accuracy (59). Therefore, to accurately predict ecosystem functioning, more studies need to be conducted to optimize methods, select appropriate functional predictors, reduce skewed sample distribution, decrease multiple incidences of collinearity, and/or increase the reliability of ecosystem functional process data.

### Conclusions.

Our results indicated that the overall functional richness/diversity decreased with increased uranium (but not nitrate) concentrations or at low or high pHs. Some specific functional genes/populations were stimulated under high concentrations of uranium or nitrate and could be used to successfully predict uranium and nitrate contamination and, potentially, ecosystem functioning. This study provides new insights for our understanding of the impacts of environmental contaminants on the functional richness/diversity of groundwater microbiomes and demonstrates the predictive power of microbial functional genes to identify environmental contamination and ecosystem functioning.

## MATERIALS AND METHODS

More detailed descriptions of the site, sampling methods, physical, geochemical and microbiological measurements, groundwater biomass collection, DNA extraction, and random forest analysis was provided previously (22).

### Site description and sampling.

The U.S. Department of Energy’s (DOE) Oak Ridge Integrated Field Research Challenge (OR-IFRC) site has a 243-acre contaminated area and a 402-acre uncontaminated background area located within the Bear Creek Valley watershed in Oak Ridge, TN. This site has been contaminated with radionuclides (e.g., uranium and technetium), nitrate, sulfide, and volatile organic compounds. The major source of contamination is the former S-3 waste disposal ponds within the Y-12 national security complex, which has been continuously monitored and documented over the past several decades (25, 60). Further information regarding the plume and sources of contamination can be found at https://public.ornl.gov/orifc/orfrc1_fieldchallenge.cfm.

### Physical, geochemical, and microbiological measurements.

In this study, 93 groundwater wells were carefully selected to cover the maximum geochemical diversity of this site without exhaustively sampling all available wells. However, we were only able to obtain enough DNA from 69 wells for GeoChip analysis (see Table S1 in the supplemental material). Groundwater samples were collected from the OR-IFRC experimental site between November 2012 and February 2013. A variety of physical, geochemical, and microbiological properties were measured on site or in the laboratory as previously described (22); a brief summary follows. (i) Bulk water parameters, including temperature, pH, dissolved oxygen (DO), conductivity, and redox, were measured at the wellhead using an In-Situ Troll 9500 sensor (In-Situ, Inc., Fort Collins, CO). (ii) Dissolved gases, including He, H_2_, N_2_, O_2_, CO, CO_2_, CH_4_, and N_2_O, were measured on an SRI 8610C gas chromatograph with argon carrier gas using a method derived from EPA RSK-175 and USGS Reston Chlorofluorocarbon Laboratory procedures. (iii) Dissolved organic carbon (DOC) and inorganic carbon (DIC) concentrations were determined with a Shimadzu TOC-V CSH analyzer (Tokyo, Japan). (iv) Anions, including bromide, chloride, nitrate, phosphate, and sulfate, were determined using a Dionex 2100 with an AS9 column and carbonate eluent. (v) Concentrations of metals (and trace elements) in the groundwater were determined on an inductively coupled plasma-mass spectrometry (ICP-MS) instrument (Elan 6100) (61). Finally, (vi) the amounts of bacterial biomass in groundwater samples were determined using the acridine orange direct count (AODC) method (62).

### Groundwater biomass collection, DNA extraction, and template preparation.

Microbial biomass was collected and DNA extracted as described previously (11). Briefly, 4.0 liters of groundwater was filtered through 0.2-µm filters to collect biomass. Filters containing biomass were placed into 50-ml Falcon tubes, immediately stored on dry ice, transferred to the laboratory, and stored at −80°C until DNA extraction. DNA was extracted and purified using a modification of the Miller method (62).

### GeoChip hybridization and data preprocessing.

The GeoChip 5.0 microarray chip contains 167,044 distinct functional gene probes, covering 395,894 coding sequences (CDS) from ~1,600 functional gene families involved in microbial carbon (e.g., degradation, methane metabolism, and fixation) and nitrogen (e.g., nitrification, denitrification, reduction, and fixation) cycling, electron transfer, organic remediation, secondary metabolism, stress responses, and virulence. To obtain sufficient DNA for microarray analysis, 10 ng of template DNA from each sample was amplified using whole-community genome amplification (WCGA) (63). After amplification, 2.5 μg of DNA was labeled, resuspended in hybridization buffer, and hybridized on a GeoChip 5.0 microarray chip with 10% formamide at 67°C for 24 h in an Agilent microarray hybridization oven (Agilent Technologies, Santa Clara, CA). The array was then washed, dried, and scanned at 100% laser power at wavelengths of 532 nm and 635 nm. Intensity data were collected using the Agilent Feature Extraction program. Raw intensity data were uploaded to the Functional Gene Microarray analysis pipeline (http://ieg2.ou.edu/Agilent) for preprocessing, including normalization and log transformation.

### GeoChip data analysis.

The preprocessed GeoChip data and environmental variables were used for further statistical analyses, including (i) α diversity and evenness indexes of microbial communities as previously described (16), (ii) linear and nonlinear regressions between measures of functional gene diversity/abundances of selected genes and geochemical properties by SigmaPlot (Systat Software, Inc., San Jose, CA), and (iii) linear regressions between each probe (normalized signal intensity profile across all samples) and environmental variables and calculations of slopes and *R*^2^ and *P* values using R (64).

### Random forest for predicting environmental contamination and ecosystem functioning.

Random forest was used for classification and regression as it does not require extensive tuning and recent studies have demonstrated that it is a suitable tool in microbial community analysis (22, 58, 65). This method included three major steps: feature selection, modeling (classification or regression), and error rate estimation by out-of-bag (OOB) data.

### (i) Feature selection.

Different sets of functional genes were selected as features for predicting environmental (uranium and nitrate) contamination and ecosystem functioning (e.g., N_2_O), including related functional gene categories (e.g., all N cycling genes), specific functional gene families (e.g., *norB* or *nosZ*), and key functional genes that were significantly increased or decreased as contamination increased. For the classification of environmental (uranium and nitrate) contamination, we also used the receiver operating characteristic curve and the area under the curve (AUC) as the predictive accuracy for random forest (RF) and then selected the set of features with the highest AUC values, termed AUC-RF (31), thus reducing the multiple collinearity among features. An AUC of around 0.5 indicates that the classification is only as good as a random guess, while the classification is perfect if the AUC is 1.0. This was performed by using the R package AUCRF.

### (ii) Modeling.

The random forest models were constructed using the R package “randomForest” as described by Leo Breiman (66). The algorithm is briefly summarized below. First, bootstrap samples were drawn from the original data *n* times. Second, for each set of bootstrap samples, an unpruned classification or regression tree was grown, and at each node, rather than choosing the best split among all features, we randomly sampled the m_try_ (number of features randomly sampled as candidates at each split) of the features and chose the best split among those features. By default, m_try_ equals one-third the number of all features. Third, new data were predicted by aggregating the predictions of *n* trees (i.e., majority votes for classification and averages for regression).

### (iii) Error rate estimation.

The estimate of the error rate was obtained without independent test data sets. At each bootstrap iteration, the data not included in the bootstrap samples, also known as out-of-bag (OOB) data, were used for prediction with the tree constructed from the bootstrap samples. Then, the error rate was calculated by aggregating the OOB predictions to obtain the OOB estimate of error rate.
