# Supplementary material for: Microbial Functional Gene Diversity Predicts Groundwater Contamination and Ecosystem Functioning
Source: mBio. 2018 Feb 20;9(1):e02435-17. doi: 10.1128/mBio.02435-17 (PMC5821090; doi:10.1128/mBio.02435-17)
Supplement: TABLE S1 [file mbo001183730st1.docx]

**Table S1** Key geochemical and ecosystem data for 69 wells selected.

| Well ID | U (mg/L) | pH | NO3- (mg/L) | CO2 (mg/L) | CH4 (mg/L) | N2O (mg/L) | Sulfide (mg/L) | AODC (Cells/ml) | DOC (mg/L) | DIC (mg/L) |
| --- | --- | --- | --- | --- | --- | --- | --- | --- | --- | --- |
| DP16D | 0.74 | 6.7 | 141.02 | 5.69 | 0.02 | 0.00 | 0.00 | 6.92E+04 | 2.33 | 87.91 |
| FW-021 | 3.75 | 3.4 | 4506.90 | 8.39 | 0.00 | 0.87 | 0.00 | 2.23E+05 | 7.30 | 22.27 |
| FW-104 | 8.99 | 5.2 | 9068.70 | 19.27 | 0.00 | 1.16 | 0.00 | 2.20E+05 | 6.51 | 117.00 |
| FW-106-2 | 16.62 | 3.6 | 2692.04 | 17.51 | 0.00 | 0.63 | 0.00 | 1.78E+05 | 47.87 | 43.18 |
| FW-126-2 | 55.29 | 3.0 | 11648.30 | 20.11 | 0.00 | 0.48 | 0.04 | 1.22E+05 | 128.20 | 36.65 |
| FW-215 | 1.45 | 6.6 | 5.50 | 4.86 | 0.01 | 0.00 | 0.00 | 3.95E+04 | 1.93 | 85.25 |
| FW-233-17-2 | 1.31 | 6.4 | 5.03 | 3.71 | 0.06 | 0.00 | 0.00 | 3.36E+04 | 4.11 | 80.73 |
| FW-300 | 0.22 | 6.6 | 3.66 | 0.00 | 0.00 | 0.00 | 0.03 | 2.74E+04 | 44.54 | 48.13 |
| FW-301-1 | 0.03 | 6.1 | 18.95 | 0 | 0 | 0 | 0.04 | 1.12E+05 | 11.58 | 41.69 |
| FW-301-2 | 0.16 | 6.7 | 36.36 | 0 | 0 | 0 | 0.19 | 1.12E+05 | 48.65 | 55.44 |
| FW-301-3 | 0.02 | 5.4 | 0.00 | 1.69 | 0.00 | 0.00 | 0.00 | 1.12E+05 | 0.61 | 15.22 |
| FW-303-1 | 0.08 | 7.2 | 3.96 | 0 | 0 | 0 | 0.04 | 2.74E+04 | 39.59 | 40.82 |
| FW-303-2 | 0.02 | 7.3 | 0.41 | 1.34 | 0.00 | 0.00 | 0.00 | 2.74E+04 | 0.55 | 39.28 |
| FW-410-28 | 13.02 | 3.7 | 1516.13 | 5.04 | 0.00 | 0.31 | 0.02 | 1.23E+05 | 2.11 | 11.49 |
| FW-510 | 0.85 | 4.0 | 850.47 | 7.36 | 0.00 | 0.27 | 0.13 | 1.05E+06 | 1.78 | 16.63 |
| FW-602-2-26 | 0.05 | 6.5 | 816.56 | 2.65 | 0.00 | 0.11 | 0.00 | 7.35E+04 | 1.60 | 51.73 |
| GW-056 | 0.01 | 6.9 | 2.56 | 1.38 | 0.00 | 0.00 | 0.03 | 2.27E+04 | 0.74 | 47.57 |
| GW-057 | 0.00 | 6.7 | 0.00 | 5.22 | 0.00 | 0.00 | 0.01 | 1.13E+04 | 1.36 | 98.66 |
| GW-066 | 0.02 | 6.2 | 0.86 | 0.89 | 0.01 | 0.00 | 0.03 | 1.83E+06 | 3.21 | 42.97 |
| GW-086 | 0.02 | 6.5 | 26.97 | 7.48 | 0.00 | 0.00 | 0.01 | 2.95E+04 | 0.40 | 47.63 |
| GW-098 | 0.02 | 6.4 | 0.00 | 12.16 | 0.01 | 0.00 | 0.00 | 1.03E+04 | 4.96 | 171.20 |
| GW-101-1 | 0.15 | 7.2 | 6.91 | 0 | 0 | 0 | 0.11 | 3.33E+05 | 40.45 | 126.90 |
| GW-101-2 | 0.42 | 6.8 | 1470.90 | 7.83 | 0.03 | 0.07 | 0.00 | 5.86E+04 | 4.07 | 115.90 |
| GW-107 | 0.01 | 6.2 | 84.13 | 4.41 | 0.00 | 0.00 | 0.03 | 7.75E+04 | 1.55 | 110.40 |
| GW-122 | 0.04 | 6.6 | 420.63 | 6.40 | 0.00 | 0.48 | 0.07 | 4.94E+04 | 0.87 | 110.70 |
| GW-123 | 0.03 | 10.0 | 1.64 | 0.00 | 0.27 | 0.00 | 0.03 | 2.75E+05 | 2.19 | 175.90 |
| GW-125 | 0.00 | 10.0 | 2.23 | 0.00 | 0.00 | 0.00 | 0.00 | 2.72E+05 | 2.05 | 179.20 |
| GW-151 | 0.01 | 7.1 | 3.69 | 1.33 | 0.01 | 0.00 | 0.00 | 6.50E+04 | 0.40 | 56.21 |
| GW-162 | 0.02 | 8.2 | 0.00 | 0.00 | 0.02 | 0.00 | 0.08 | 2.07E+05 | 0.46 | 29.26 |
| GW-198 | 0.00 | 6.1 | 5.83 | 2.91 | 0.00 | 0.00 | 0.02 | 5.27E+04 | 0.86 | 30.18 |
| GW-199 | 0.00 | 6.5 | 0.17 | 4.81 | 0.00 | 0.00 | 0.00 | 4.44E+04 | 1.34 | 85.05 |
| GW-220 | 0.00 | 7.5 | 2.07 | 2.31 | 0.00 | 0.00 | 0.00 | 3.71E+04 | 0.39 | 57.34 |
| GW-223 | 0.04 | 6.8 | 0.00 | 1.51 | 0.00 | 0.00 | 0.00 | 1.06E+03 | 1.16 | 52.64 |
| GW-228 | 0.01 | 9.3 | 7.62 | 0.00 | 0.03 | 0.00 | 0.01 | 9.50E+04 | 0.77 | 11.56 |
| GW-271 | 0.00 | 7.7 | 0.00 | 1.78 | 0.00 | 0.00 | 4.15 | 4.18E+04 | 0.59 | 49.23 |
| GW-283 | 0.02 | 7.0 | 3.14 | 4.23 | 0.00 | 0.00 | 0.01 | 5.86E+04 | 2.71 | 88.28 |
| GW-345 | 0.02 | 5.2 | 171.02 | 3.38 | 0.00 | 0.18 | 0.00 | 4.21E+04 | 0.33 | 18.31 |
| GW-346 | 0.02 | 6.6 | 4590.63 | 0.44 | 0.00 | 0.39 | 0.00 | 7.54E+04 | 0.51 | 12.57 |
| GW-350 | 0.01 | 6.7 | 0.00 | 3.59 | 0.00 | 0.00 | 0.00 | 3.54E+02 | 0.72 | 67.46 |
| GW-363 | 0.05 | 9.1 | 0.42 | 0.00 | 0.00 | 0.00 | 0.01 | 1.65E+04 | 0.44 | 58.43 |
| GW-383 | 0.00 | 7.1 | 0.10 | 6.47 | 0.00 | 0.00 | 0.00 | 3.10E+04 | 0.38 | 49.79 |
| GW-385 | 0.00 | 9.2 | 0.28 | 0.00 | 0.00 | 0.00 | 0.00 | 4.91E+04 | 0.78 | 88.14 |
| GW-526 | 0.01 | 8.0 | 5849.96 | 0.00 | 0.00 | 0.24 | 0.01 | 8.13E+04 | 2.47 | 9.38 |
| GW-60-1A | 0.00 | 7.4 | 0.19 | 1.99 | 0.01 | 0.00 | 0.03 | 1.39E+04 | 0.91 | 48.81 |
| GW-621 | 0.02 | 7.2 | 7.63 | 1.95 | 0.00 | 0.00 | 0.00 | 3.90E+04 | 0.98 | 49.18 |
| GW-631 | 0.01 | 4.3 | 1.40 | 6.16 | 0.04 | 0.00 | 0.00 | 1.18E+05 | 1.79 | 27.58 |
| GW-636 | 0.01 | 8.9 | 0.14 | 0.00 | 0.02 | 0.00 | 0.23 | 5.29E+05 | 0.37 | 42.92 |
| GW-654 | 0.02 | 7.2 | 0.46 | 1.01 | 0.00 | 0.00 | 0.00 | 4.99E+04 | 0.35 | 30.98 |
| GW-658 | 0.00 | 6.4 | 0.00 | 6.47 | 0.00 | 0.00 | 0.00 | 1.67E+05 | 68.37 | 61.69 |
| GW-685 | 0.00 | 6.9 | 1.56 | 4.92 | 0.00 | 0.00 | 0.01 | 1.54E+04 | 0.92 | 85.60 |
| GW-694 | 0.03 | 7.3 | 19.48 | 1.66 | 0.00 | 0.00 | 0.00 | 1.12E+05 | 1.18 | 44.12 |
| GW-705 | 0.00 | 9.2 | 0.00 | 0.00 | 0.04 | 0.00 | 1.89 | 6.59E+04 | 0.88 | 69.31 |
| GW-706 | 0.05 | 7.9 | 75.72 | 6.44 | 0.00 | 0.00 | 0.00 | 6.19E+04 | 1.17 | 74.33 |
| GW-714 | 0.01 | 7.5 | 2.33 | 2.38 | 0.00 | 0.00 | 0.00 | 4.23E+04 | 0.23 | 49.72 |
| GW-715 | 0.01 | 6.7 | 3.32 | 3.64 | 0.00 | 0.00 | 0.00 | 2.01E+03 | 0.79 | 47.94 |
| GW-736 | 0.04 | 6.6 | 28.84 | 3.87 | 0.00 | 0.00 | 0.04 | 4.99E+04 | 1.17 | 73.42 |
| GW-753 | 0.00 | 10.5 | 0.26 | 29739 | 0.00 | 0.00 | 0.66 | 2.48E+03 | 0.36 | 29.55 |
| GW-761 | 0.00 | 6.6 | 10.64 | 2.18 | 0.00 | 0.00 | 0.00 | 2.48E+04 | 0.31 | 31.00 |
| GW-763 | 0.00 | 6.7 | 0.00 | 4.21 | 0.65 | 0.00 | 0.01 | 2.62E+04 | 2.09 | 76.24 |
| GW-764 | 0.15 | 8.6 | 14.43 | 15.83 | 0.00 | 0.00 | 0.00 | 6.24E+04 | 1.22 | 35.55 |
| GW-775 | 0.01 | 7.2 | 5.02 | 2.21 | 0.00 | 0.00 | 0.00 | 5.43E+03 | 1.02 | 51.20 |
| GW-779 | 0.00 | 9.6 | 0.00 | 0.00 | 0.00 | 0.00 | 0.00 | 1.06E+03 | 0.51 | 41.65 |
| GW-800 | 0.00 | 6.7 | 0.00 | 2.59 | 0.00 | 0.00 | 0.00 | 1.98E+04 | 0.60 | 52.15 |
| GW-803 | 0.00 | 5.4 | 33.89 | 2.44 | 0.00 | 0.00 | 0.00 | 1.56E+04 | 0.31 | 16.07 |
| GW-804 | 0.00 | 4.9 | 10.81 | 2.02 | 0.00 | 0.00 | 0.09 | 6.52E+04 | 0.54 | 15.85 |
| GW-925 | 0.02 | 9.8 | 2.27 | 0.00 | 0.00 | 0.00 | 0.06 | 8.60E+04 | 1.39 | 55.53 |
| GW-928 | 0.00 | 6.7 | 0.00 | 2.02 | 0.00 | 0.00 | 0.00 | 3.12E+04 | 0.46 | 29.88 |
| GW-929 | 0.00 | 5.2 | 0.18 | 1.83 | 0.00 | 0.00 | 0.02 | 3.26E+04 | 0.36 | 9.71 |
| TPB16 | **1.15** | **7.1** | **25.07** | **0** | **0** | **0** | **0.01** | **5.22E+04** | **23.78** | **91.49** |
| Average | **1.52** | **6.87** | **640.72** | **468.61** | **0.02** | **0.08** | **0.12** | **117465.51** | **7.79** | **58.34** |
| Minimum | **0.00** | **3.04** | **0.00** | **0.00** | **0.00** | **0.00** | **0.00** | **354.44** | **0.23** | **9.38** |
| Maximum | **55.29** | **10.48** | **11648.0** | **29739.1** | **0.65** | **1.16** | **4.15** | **1827347** | **128.20** | **179.20** |
