# Supplementary figures and images for: Microbial Functional Gene Diversity Predicts Groundwater Contamination and Ecosystem Functioning
Source: mBio. 2018 Feb 20;9(1):e02435-17. doi: 10.1128/mBio.02435-17 (PMC5821090; doi:10.1128/mBio.02435-17)

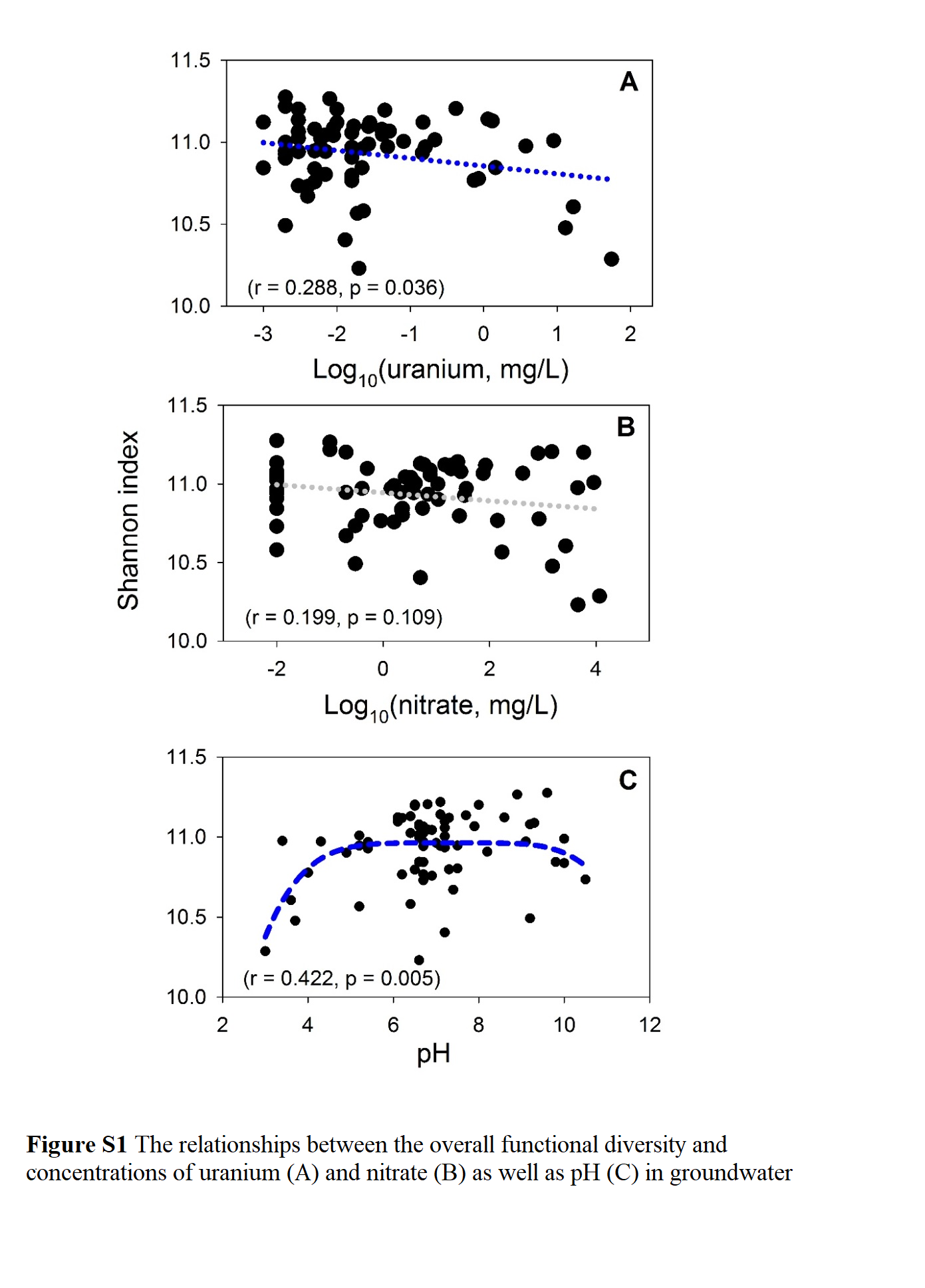

Supplement: FIG S1 [file mbo001183730sf1.tif]

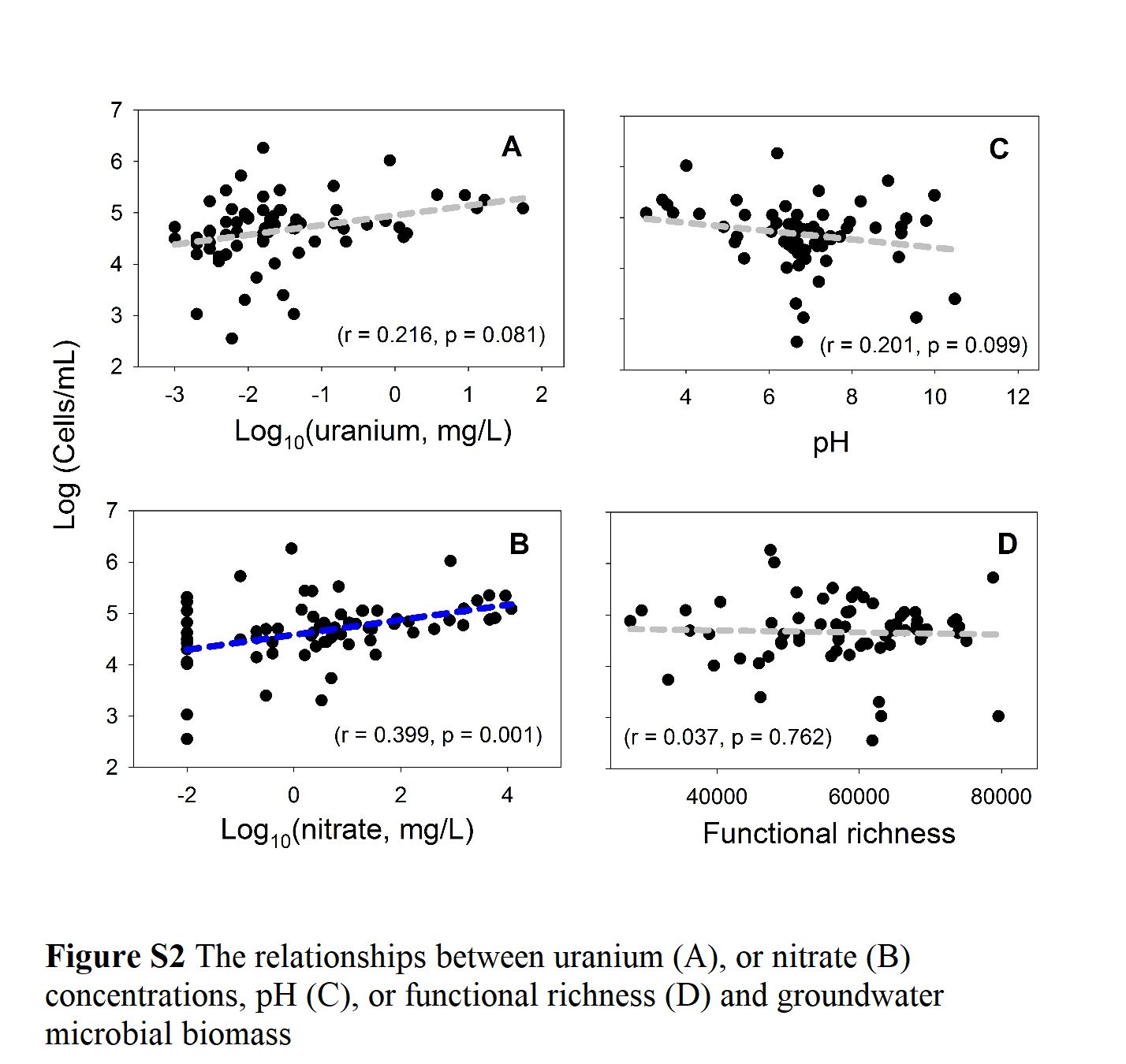

Supplement: FIG S2 [file mbo001183730sf2.tif]
