# Supplementary material for: Microbial Functional Gene Diversity Predicts Groundwater Contamination and Ecosystem Functioning
Source: mBio. 2018 Feb 20;9(1):e02435-17. doi: 10.1128/mBio.02435-17 (PMC5821090; doi:10.1128/mBio.02435-17)
Supplement: TABLE S2 [file mbo001183730st2.docx]

**Table S2** Regressions of key nitrogen cycling gene abundance with uranium, nitrate, or pH. Uranium and nitrate concentrations were first log-transformed, and then used for linear regressions, while non-linear regression was used for pH without log-transformation. P values < 0.05 are bold.

| Functional gene | Uranium concentration | | Nitrate concentration | | pH | |
| --- | --- | --- | --- | --- | --- | --- |
|  | *r* | *p* | *r* | *p* | *r* | *p* |
| *narG* | 0.389 | **0.011** | 0.225 | 0.072 | 0.369 | **0.015** |
| *nirK* | 0.316 | **0.025** | 0.161 | 0.187 | 0.388 | **0.014** |
| *nirS* | 0.325 | **0.026** | 0.182 | 0.138 | 0.484 | **0.018** |
| *norB* | 0.289 | **0.033** | 0.196 | 0.106 | 0.353 | **0.016** |
| *nosZ* | 0.311 | **0.023** | 0.125 | 0.301 | 0.354 | **0.033** |
| *nasA* | 0.328 | **0.021** | 0.210 | 0.083 | 0.399 | **0.010** |
| *napA* | 0.301 | **0.034** | 0.126 | 0.304 | 0.367 | **0.024** |
| *nrfA* | 0.328 | **0.015** | 0.203 | 0.094 | 0.322 | **0.029** |
| *nifH* | 0.292 | **0.033** | 0.208 | 0.115 | 0.469 | **0.006** |
| *amoA* | 0.274 | **0.044** | 0.160 | 0.189 | 0.364 | **0.023** |
