# Supplementary material for: Microbial Functional Gene Diversity Predicts Groundwater Contamination and Ecosystem Functioning
Source: mBio. 2018 Feb 20;9(1):e02435-17. doi: 10.1128/mBio.02435-17 (PMC5821090; doi:10.1128/mBio.02435-17)
Supplement: TABLE S3 [file mbo001183730st3.docx]

**Table S3** Relationships between the abundance of significantly increased or decreased populations (key genes) and uranium concentrations by a linear regression. Significantly increased slopes are bold, and the relative abundances are presented in mean ratio.

| GenBank ID | Organism | Slope | P-value | R^2^ | Mean ratio |
| --- | --- | --- | --- | --- | --- |
| ***dsrA*** | | | | | |
| 40736909 | Uncultured bacterium | **3.690** | 0.012 | 0.566 | 0.282 |
| 109452512 | Uncultured sulfate-reducing bacterium | **2.594** | 0.001 | 0.958 | 0.161 |
| 308152084 | Uncultured sulfate-reducing bacterium | **1.767** | 0.013 | 1.000 | 0.082 |
| 84778307 | Uncultured sulfate-reducing bacterium | **1.469** | 0.001 | 0.532 | 0.432 |
| 350532577 | *Vibrio rotiferianus* | **0.972** | 0.004 | 0.488 | 0.401 |
| 46519955 | Uncultured sulfate-reducing bacterium | **0.400** | 0.000 | 0.457 | 0.713 |
| 13560124 | *Thermodesulforhabdus norvegica* | **0.319** | 0.001 | 0.956 | 0.168 |
| 70930919 | Uncultured sulfate-reducing bacterium | **0.318** | 0.001 | 0.561 | 0.457 |
| 46520001 | Uncultured sulfate-reducing bacterium | **0.254** | 0.019 | 0.257 | 0.553 |
| 157679419 | Uncultured sulfate-reducing bacterium | **0.220** | 0.002 | 0.971 | 0.138 |
| 45357641 | *Methanococcus maripaludis* | **0.150** | 0.000 | 0.721 | 0.340 |
| 227438049 | Uncultured bacterium | **0.147** | 0.037 | 0.220 | 0.551 |
| 313105092 | Uncultured prokaryote | **0.135** | 0.009 | 0.547 | 0.296 |
| 109452449 | Uncultured sulfate-reducing bacterium | **0.113** | 0.016 | 0.150 | 1.050 |
| 341834350 | Uncultured prokaryote | **0.107** | 0.044 | 0.417 | 0.270 |
| 56566220 | Uncultured sulfate-reducing bacterium | **0.084** | 0.047 | 0.669 | 0.158 |
| 208936504 | Uncultured prokaryote | **0.075** | 0.001 | 0.479 | 0.592 |
| 374413696 | Uncultured prokaryote | **0.056** | 0.038 | 0.229 | 0.529 |
| 163929795 | *Desulfovibrionaceae bacterium* | **0.051** | 0.048 | 0.905 | 0.104 |
| 82618405 | Uncultured sulfate-reducing bacterium | **0.040** | 0.042 | 0.687 | 0.165 |
| 225194046 | Uncultured sulfate-reducing bacterium | **0.038** | 0.035 | 0.095 | 1.302 |
| 218142839 | *Desulfovibrio salexigens* | **0.036** | 0.024 | 0.756 | 0.159 |
| 46307974 | Uncultured sulfate-reducing bacterium | **0.032** | 0.001 | 0.189 | 1.744 |
| 78165828 | *Pelodictyon luteolum* | **0.029** | 0.016 | 0.258 | 0.590 |
| 144905794 | Uncultured sulfate-reducing bacterium | **0.026** | 0.017 | 0.886 | 0.141 |
| 157679477 | Uncultured sulfate-reducing bacterium | **0.024** | 0.046 | 0.412 | 0.275 |
| 121590136 | *Halorhodospira halophila* | **0.019** | 0.004 | 0.203 | 1.072 |
| 33320480 | Sulfate-reducing bacterium | **0.019** | 0.038 | 0.926 | 0.112 |
| 320355026 | *Desulfobulbus propionicus* | **0.018** | 0.003 | 0.334 | 0.664 |
| 118424508 | Uncultured sulfate-reducing bacterium | **0.018** | 0.000 | 0.932 | 0.187 |
| 40253076 | Uncultured sulfate-reducing bacterium | **0.013** | 0.009 | 0.418 | 0.395 |
| 12667676 | Uncultured sulfate-reducing bacterium | **0.012** | 0.004 | 0.356 | 0.547 |
| 46307833 | Uncultured sulfate-reducing bacterium | **0.009** | 0.045 | 0.344 | 0.319 |
| 237846130 | Uncultured sulfate-reducing bacterium | **0.005** | 0.001 | 0.180 | 1.560 |
| 84778391 | Uncultured sulfate-reducing bacterium | **0.004** | 0.004 | 0.309 | 0.671 |
| 46307906 | Uncultured sulfate-reducing bacterium | **0.003** | 0.006 | 0.313 | 0.613 |
| 46307829 | Uncultured sulfate-reducing bacterium | **0.003** | 0.008 | 0.276 | 0.639 |
| 46307916 | Uncultured sulfate-reducing bacterium | **0.003** | 0.017 | 0.966 | 0.104 |
| 46308012 | Uncultured sulfate-reducing bacterium | **0.002** | 0.012 | 0.124 | 1.384 |
| 46307852 | Uncultured sulfate-reducing bacterium | **0.002** | 0.040 | 0.152 | 0.759 |
| 37726843 | Uncultured prokaryote | **0.002** | 0.032 | 0.091 | 1.425 |
| 157679062 | Uncultured sulfate-reducing bacterium | **0.002** | 0.016 | 0.155 | 0.984 |
| 46307858 | Uncultured sulfate-reducing bacterium | **0.002** | 0.035 | 0.082 | 1.471 |
| 126249775 | *Pyrobaculum calidifontis* | -0.002 | 0.039 | 0.062 | 2.680 |
| 229473039 | Uncultured bacterium | -0.002 | 0.021 | 0.077 | 2.281 |
| 90954635 | Uncultured sulfate-reducing bacterium | -0.002 | 0.003 | 0.121 | 2.291 |
| 37726855 | Uncultured prokaryote | -0.002 | 0.012 | 0.091 | 2.433 |
| 84778339 | Uncultured sulfate-reducing bacterium | -0.003 | 0.001 | 0.160 | 2.220 |
| 56694506 | Uncultured sulfate-reducing bacterium | -0.003 | 0.047 | 0.059 | 2.290 |
| 313105036 | Uncultured prokaryote | -0.003 | 0.031 | 0.069 | 2.110 |
| 237846178 | Uncultured sulfate-reducing bacterium | -0.003 | 0.009 | 0.099 | 2.358 |
| 90954569 | Uncultured sulfate-reducing bacterium | -0.004 | 0.000 | 0.182 | 2.355 |
| 371486302 | Uncultured sulfate-reducing bacterium | -0.006 | 0.000 | 0.221 | 2.537 |
| 158523904 | Uncultured bacterium | -0.007 | 0.000 | 0.201 | 2.548 |
| 18034316 | *Syntrophobacter wolinii* | -0.009 | 0.014 | 0.089 | 2.176 |
| 88062453 | Uncultured bacterium | -0.010 | 0.006 | 0.110 | 2.199 |
| 56566208 | Uncultured sulfate-reducing bacterium | -0.010 | 0.013 | 0.091 | 2.120 |
| 158523640 | Uncultured bacterium | -0.011 | 0.017 | 0.086 | 2.200 |
| 158523516 | Uncultured bacterium | -0.011 | 0.002 | 0.142 | 2.231 |
| 109452551 | Uncultured sulfate-reducing bacterium | -0.011 | 0.004 | 0.123 | 2.178 |
| 118424457 | Uncultured sulfate-reducing bacterium | -0.011 | 0.042 | 0.064 | 2.000 |
| 222477096 | Uncultured bacterium | -0.012 | 0.001 | 0.146 | 2.178 |
| 46520023 | Uncultured sulfate-reducing bacterium | -0.013 | 0.001 | 0.150 | 2.430 |
| 84778347 | Uncultured sulfate-reducing bacterium | -0.016 | 0.010 | 0.098 | 2.297 |
| 209171678 | *Candidatus Thiobios zoothamnicoli* | -0.018 | 0.000 | 0.206 | 2.301 |
| 20142110 | Uncultured bacterium | -0.048 | 0.025 | 0.408 | 0.312 |
| 84778337 | Uncultured sulfate-reducing bacterium | -7.784 | 0.040 | 0.428 | 0.280 |
| **Cytochrome genes** | | | | | |
| 253699332 | *Geobacter sp.* | **0.240** | 0.019 | 0.999 | 0.079 |
| 157373623 | *Shewanella sediminis* | **0.177** | 0.015 | 0.897 | 0.124 |
| 296031293 | *Thermincola potens* | **0.159** | 0.001 | 0.661 | 0.284 |
| 119775535 | *Shewanella amazonensis* | **0.136** | 0.025 | 0.277 | 0.454 |
| 71848494 | *Dechloromonas aromatica* | **0.122** | 0.041 | 0.600 | 0.174 |
| 146307224 | *Pseudomonas mendocina* | **0.113** | 0.033 | 0.559 | 0.197 |
| 336248772 | *Enterobacter aerogenes* | **0.102** | 0.000 | 0.362 | 1.020 |
| 157374541 | *Shewanella sediminis* | **0.069** | 0.050 | 0.994 | 0.072 |
| 119775363 | *Shewanella amazonensis* | **0.068** | 0.003 | 0.431 | 0.459 |
| 119773387 | *Shewanella amazonensis* | **0.066** | 0.005 | 0.469 | 0.387 |
| 91794915 | *Shewanella denitrificans* | **0.061** | 0.000 | 0.803 | 0.379 |
| 70733596 | *Pseudomonas fluorescens* | **0.024** | 0.000 | 0.554 | 0.986 |
| 127513453 | *Shewanella loihica* | **0.018** | 0.017 | 0.208 | 0.680 |
| 157375053 | *Shewanella sediminis* | **0.017** | 0.014 | 0.134 | 1.138 |
| 39995725 | *Geobacter sulfurreducens* | **0.012** | 0.003 | 0.960 | 0.121 |
| 71847730 | *Dechloromonas aromatica* | **0.011** | 0.043 | 0.143 | 0.708 |
| 148262414 | *Geobacter uraniumreducens* | **0.010** | 0.050 | 0.112 | 0.846 |
| 394728887 | *Enterobacter sp.* | **0.003** | 0.009 | 0.111 | 1.590 |
| 393759946 | *Alcaligenes faecalis* | **0.002** | 0.027 | 0.071 | 2.031 |
| 254982574 | *Geobacter sp.* | **0.002** | 0.022 | 0.079 | 1.871 |
| 384085650 | *Acidithiobacillus thiooxidans* | **0.001** | 0.021 | 0.426 | 0.294 |
| 218758428 | *Desulfovibrio vulgaris* | -0.002 | 0.043 | 0.060 | 1.940 |
| 393164934 | *Alcaligenes faecalis* | -0.002 | 0.043 | 0.060 | 2.054 |
| 146279248 | *Rhodobacter sphaeroides* | -0.003 | 0.016 | 0.084 | 2.278 |
| 78355627 | *Desulfovibrio desulfuricans* | -0.003 | 0.029 | 0.070 | 2.196 |
| 239833566 | *Ochrobactrum intermedium* | -0.003 | 0.012 | 0.093 | 2.031 |
| 393759947 | *Alcaligenes faecalis* | -0.003 | 0.019 | 0.079 | 2.326 |
| 393162856 | *Alcaligenes faecalis* | -0.003 | 0.003 | 0.123 | 2.095 |
| 395235190 | *Enterobacter sp.* | -0.004 | 0.006 | 0.106 | 2.220 |
| 146277090 | *Rhodobacter sphaeroides* | -0.004 | 0.001 | 0.141 | 2.113 |
| 167034533 | *Pseudomonas putida* | -0.005 | 0.001 | 0.141 | 2.224 |
| 86159495 | *Anaeromyxobacter dehalogenans* | -0.005 | 0.000 | 0.190 | 2.242 |
| 146277744 | *Rhodobacter sphaeroides* | -0.006 | 0.000 | 0.185 | 2.185 |
| 340782716 | *Acidithiobacillus caldus* | -0.006 | 0.000 | 0.213 | 2.319 |
| 254985782 | *Geobacter sp.* | -0.007 | 0.047 | 0.060 | 2.059 |
| 153009994 | *Ochrobactrum anthropi* | -0.010 | 0.010 | 0.096 | 2.048 |
| 218885637 | *Desulfovibrio vulgaris* | -0.010 | 0.004 | 0.120 | 1.906 |
| 127511457 | *Shewanella loihica* | -0.012 | 0.032 | 0.070 | 1.980 |
| 212558076 | *Shewanella piezotolerans* | -0.099 | 0.001 | 0.985 | 0.126 |
| 157375843 | *Shewanella sediminis* | -1.041 | 0.025 | 0.998 | 0.073 |
| 157960332 | *Shewanella pealeana* | -1.752 | 0.016 | 0.999 | 0.078 |
| 78223673 | *Geobacter metallireducens* | -2.177 | 0.047 | 0.995 | 0.073 |
| 166861022 | *Pseudomonas putida* | -18.823 | 0.008 | 1.000 | 0.083 |
| **Hydrogenase genes** | | | | | |
| 218758321 | *Desulfovibrio vulgaris* | **0.051** | 0.021 | 0.616 | 0.172 |
| 24373658 | *Shewanella oneidensis* | **0.020** | 0.003 | 0.386 | 0.440 |
| 78217815 | *Desulfovibrio desulfuricans* | **0.009** | 0.038 | 0.080 | 1.163 |
| 6466828 | *Desulfitobacterium dehalogenans* | **0.008** | 0.028 | 0.284 | 0.357 |
| 218142115 | *Desulfovibrio salexigens* | **0.003** | 0.006 | 0.690 | 0.191 |
| 242122281 | *Desulfovibrio salexigens* | **0.003** | 0.043 | 0.680 | 0.130 |
| 239796540 | *Desulfovibrio magneticus* | -0.003 | 0.016 | 0.084 | 1.748 |
| 218757779 | *Desulfovibrio vulgaris* | -0.004 | 0.001 | 0.142 | 1.818 |
| 220904110 | *Desulfovibrio desulfuricans* | -0.005 | 0.002 | 0.134 | 1.952 |
