# Supplementary material for: Microbial Functional Gene Diversity Predicts Groundwater Contamination and Ecosystem Functioning
Source: mBio. 2018 Feb 20;9(1):e02435-17. doi: 10.1128/mBio.02435-17 (PMC5821090; doi:10.1128/mBio.02435-17)
Supplement: TABLE S4 [file mbo001183730st4.docx]

**Table S4** Relationships between the abundance of significantly increased or decreased populations (key genes) and nitrate concentrations by a linear regression. Significantly increased slopes are bold, and the relative abundances are presented in mean ratio.

| Genbank ID | Organism | Slope | P-value | R^2^ | Mean ratio |
| --- | --- | --- | --- | --- | --- |
| ***nasA*** | | | | | |
| 30269563 | Uncultured bacterium | **0.749** | 0.000 | 0.683 | 0.467 |
| 30269567 | Uncultured bacterium | **0.034** | 0.013 | 0.314 | 0.435 |
| 152982608 | *Janthinobacterium sp.* | **0.027** | 0.019 | 0.963 | 0.089 |
| 140713835 | Uncultured bacterium | **0.025** | 0.003 | 0.385 | 0.468 |
| 84712443 | *Polaromonas naphthalenivorans* | **0.012** | 0.000 | 0.207 | 1.225 |
| 50085048 | *Acinetobacter sp.* | **0.011** | 0.031 | 0.213 | 0.484 |
| 84385741 | *Vibrio splendidus* | **0.010** | 0.000 | 0.201 | 1.279 |
| 140713641 | Uncultured bacterium | **0.009** | 0.039 | 0.478 | 0.200 |
| 307944736 | *Roseibium sp.* | **0.008** | 0.013 | 0.106 | 1.310 |
| 109457400 | *Roseobacter denitrificans* | -0.006 | 0.006 | 0.109 | 1.739 |
| ***narG*** | | | | | |
| 223586093 | Uncultured bacterium | **0.060** | 0.019 | 0.784 | 0.117 |
| 307131281 | *Dickeya dadantii* | **0.050** | 0.008 | 0.402 | 0.338 |
| 45386249 | Uncultured bacterium | **0.036** | 0.004 | 0.480 | 0.309 |
| 121495554 | Uncultured bacterium | **0.031** | 0.021 | 0.999 | 0.063 |
| 45386155 | Unidentified bacterium | **0.029** | 0.005 | 0.159 | 0.985 |
| 38427032 | Uncultured bacterium | **0.026** | 0.032 | 0.110 | 0.890 |
| 346644293 | Uncultured bacterium | **0.025** | 0.044 | 0.243 | 0.342 |
| 29652604 | Uncultured bacterium | **0.024** | 0.004 | 0.511 | 0.264 |
| 1911243 | *Pseudomonas fluorescens* | **0.023** | 0.045 | 0.516 | 0.158 |
| 76056949 | Uncultured bacterium | **0.023** | 0.006 | 0.161 | 0.932 |
| 209401692 | Uncultured bacterium | **0.023** | 0.036 | 0.092 | 0.992 |
| 116633914 | Uncultured bacterium | **0.019** | 0.009 | 0.514 | 0.237 |
| 66801871 | Unidentified bacterium | **0.018** | 0.001 | 0.172 | 1.242 |
| 209401856 | Uncultured bacterium | **0.018** | 0.003 | 0.133 | 1.392 |
| 209401744 | Uncultured bacterium | **0.017** | 0.027 | 0.119 | 0.859 |
| 45386193 | Unidentified bacterium | **0.016** | 0.034 | 0.082 | 1.159 |
| 134265838 | *Geobacillus thermodenitrificans* | **0.016** | 0.009 | 0.136 | 0.999 |
| 119371704 | *Castellaniella defragrans* | **0.015** | 0.003 | 0.139 | 1.395 |
| 70725655 | *Staphylococcus haemolyticus* | **0.015** | 0.032 | 0.937 | 0.077 |
| 169793464 | Uncultured bacterium | **0.014** | 0.007 | 0.104 | 1.471 |
| 325979789 | Uncultured bacterium | **0.014** | 0.016 | 0.095 | 1.246 |
| 209401868 | Uncultured bacterium | **0.013** | 0.002 | 0.163 | 1.161 |
| 26278828 | Uncultured bacterium | **0.011** | 0.014 | 0.093 | 1.361 |
| 223586018 | Uncultured bacterium | **0.010** | 0.025 | 0.310 | 0.310 |
| 306517710 | Uncultured bacterium | **0.009** | 0.007 | 0.129 | 1.112 |
| 192764372 | Uncultured bacterium | **0.008** | 0.013 | 0.090 | 1.452 |
| 32308033 | Uncultured bacterium | **0.007** | 0.022 | 0.084 | 1.255 |
| 18413621 | *Halomonas halodenitrificans* | **0.006** | 0.038 | 0.077 | 1.133 |
| 119371714 | *Thauera aromatica* | -0.008 | 0.029 | 0.069 | 1.630 |
| 94471125 | Uncultured bacterium | -0.008 | 0.028 | 0.070 | 1.673 |
| 94471271 | Uncultured bacterium | -0.008 | 0.034 | 0.065 | 1.650 |
| 29652454 | Uncultured bacterium | -0.009 | 0.003 | 0.125 | 1.690 |
| 160917463 | Uncultured bacterium | -0.009 | 0.017 | 0.082 | 1.680 |
| 26278770 | Uncultured bacterium | -0.009 | 0.016 | 0.083 | 1.724 |
| 192764352 | Uncultured bacterium | -0.010 | 0.038 | 0.064 | 1.590 |
| 192764452 | Uncultured bacterium | -0.011 | 0.011 | 0.092 | 1.716 |
| 209401654 | Uncultured bacterium | -0.011 | 0.049 | 0.057 | 1.818 |
| 209401904 | Uncultured bacterium | -0.011 | 0.019 | 0.080 | 1.810 |
| 158138854 | Uncultured bacterium | -0.011 | 0.012 | 0.090 | 1.887 |
| 94471237 | Uncultured bacterium | -0.012 | 0.008 | 0.100 | 1.595 |
| 291369137 | *Haloferax volcanii* | -0.012 | 0.012 | 0.091 | 1.752 |
| 124488241 | Uncultured bacterium | -0.012 | 0.025 | 0.072 | 1.687 |
| 119391433 | Uncultured bacterium | -0.013 | 0.013 | 0.089 | 1.722 |
| 38427020 | Uncultured bacterium | -0.013 | 0.017 | 0.082 | 1.740 |
| 306517003 | Uncultured bacterium | -0.013 | 0.001 | 0.148 | 1.979 |
| 121495608 | Uncultured bacterium | -0.014 | 0.013 | 0.089 | 1.819 |
| 209401770 | Uncultured bacterium | -0.014 | 0.042 | 0.061 | 1.749 |
| 94471177 | Uncultured bacterium | -0.014 | 0.009 | 0.096 | 1.828 |
| 119391545 | Uncultured bacterium | -0.014 | 0.016 | 0.083 | 1.795 |
| 32307925 | Uncultured bacterium | -0.014 | 0.022 | 0.076 | 1.687 |
| 306516909 | Uncultured bacterium | -0.015 | 0.001 | 0.162 | 1.810 |
| 209401594 | Uncultured bacterium | -0.016 | 0.007 | 0.104 | 1.782 |
| 62003539 | Uncultured bacterium | -0.016 | 0.006 | 0.106 | 1.917 |
| 94471103 | Uncultured bacterium | -0.016 | 0.004 | 0.115 | 1.903 |
| 192764382 | Uncultured bacterium | -0.017 | 0.001 | 0.152 | 1.958 |
| 119391597 | Uncultured bacterium | -0.019 | 0.000 | 0.185 | 1.817 |
| 116634769 | Uncultured bacterium | -0.019 | 0.001 | 0.148 | 1.838 |
| 29652532 | Uncultured bacterium | -0.020 | 0.000 | 0.213 | 1.978 |
| 209401600 | Uncultured bacterium | -0.023 | 0.000 | 0.260 | 1.948 |
| 238927773 | *Selenomonas flueggei* | -0.025 | 0.000 | 0.264 | 1.863 |
| 119391437 | Uncultured bacterium | -0.026 | 0.000 | 0.294 | 1.933 |
| ***nirK*** | | | | | |
| 104304993 | Uncultured denitrifying bacterium | **0.070** | 0.022 | 0.682 | 0.173 |
| 76577544 | Uncultured bacterium | **0.057** | 0.002 | 0.339 | 0.636 |
| 316981104 | Uncultured Proteobacterium | **0.042** | 0.010 | 0.165 | 0.960 |
| 110632256 | Uncultured bacterium | **0.022** | 0.045 | 0.256 | 0.389 |
| 116204223 | *Chaetomium globosum* | **0.017** | 0.000 | 0.309 | 1.218 |
| 73762994 | Uncultured bacterium | **0.016** | 0.013 | 0.364 | 0.367 |
| 296811482 | *Arthroderma otae* | **0.013** | 0.036 | 0.442 | 0.242 |
| 112463805 | Uncultured bacterium | **0.012** | 0.015 | 0.216 | 0.648 |
| 256723237 | *Nectria haematococca* | **0.011** | 0.009 | 0.136 | 1.177 |
| 73762878 | Uncultured bacterium | **0.010** | 0.009 | 0.128 | 1.293 |
| 46409951 | Uncultured bacterium | **0.009** | 0.039 | 0.071 | 1.509 |
| 50541845 | Uncultured bacterium | **0.008** | 0.017 | 0.092 | 1.504 |
| 68347101 | *Pseudomonas fluorescens* | **0.007** | 0.042 | 0.097 | 0.994 |
| 83317000 | Uncultured bacterium | **0.006** | 0.044 | 0.066 | 1.508 |
| 316981076 | Uncultured Proteobacterium | -0.009 | 0.040 | 0.062 | 1.898 |
| 316981166 | Uncultured Proteobacterium | -0.010 | 0.005 | 0.110 | 2.049 |
| 385699559 | *Neisseria sicca* | -0.010 | 0.010 | 0.096 | 2.109 |
| 109632645 | Uncultured bacterium | -0.011 | 0.013 | 0.089 | 2.128 |
| 225683239 | *Paracoccidioides brasiliensis* | -0.011 | 0.016 | 0.084 | 2.051 |
| 316981042 | Uncultured Proteobacterium | -0.012 | 0.005 | 0.110 | 2.244 |
| 17742853 | *Agrobacterium tumefaciens* | -0.012 | 0.005 | 0.112 | 2.421 |
| 258520125 | *Cardiobacterium hominis* | -0.015 | 0.005 | 0.112 | 2.413 |
| 86285356 | *Rhizobium etli* | -0.016 | 0.006 | 0.106 | 2.167 |
| 83316998 | Uncultured bacterium | -0.019 | 0.000 | 0.231 | 2.304 |
| 73762956 | Uncultured bacterium | -0.028 | 0.002 | 0.130 | 2.244 |
| 111072010 | Uncultured bacterium | -1.412 | 0.048 | 0.449 | 0.214 |
| 51491442 | Uncultured bacterium | -4.312 | 0.022 | 0.999 | 0.074 |
| ***nirS*** | | | | | |
| 77378707 | Uncultured bacterium | **10.347** | 0.004 | 0.834 | 0.188 |
| 77378655 | Uncultured bacterium | **9.398** | 0.008 | 0.860 | 0.155 |
| 68164660 | Uncultured bacterium | **3.137** | 0.004 | 0.992 | 0.108 |
| 237637180 | Uncultured bacterium | **0.298** | 0.046 | 0.909 | 0.108 |
| 46850256 | Uncultured bacterium | **0.197** | 0.019 | 0.701 | 0.188 |
| 76577386 | Uncultured bacterium | **0.056** | 0.032 | 0.328 | 0.402 |
| 167596212 | Uncultured bacterium | **0.046** | 0.000 | 0.705 | 0.452 |
| 330375450 | Uncultured bacterium | **0.037** | 0.002 | 0.249 | 0.946 |
| 240247506 | Uncultured bacterium | **0.033** | 0.004 | 0.959 | 0.133 |
| 304313314 | Gamma-proteobacterium | **0.028** | 0.049 | 0.077 | 1.463 |
| 355468584 | Uncultured bacterium | **0.026** | 0.013 | 0.126 | 1.346 |
| 77378641 | Uncultured bacterium | **0.023** | 0.005 | 0.416 | 0.473 |
| 340841639 | Uncultured denitrifying bacterium | **0.022** | 0.039 | 0.180 | 0.635 |
| 28542591 | Uncultured bacterium | **0.018** | 0.002 | 0.220 | 1.105 |
| 29824653 | Uncultured bacterium | **0.011** | 0.002 | 0.156 | 1.572 |
| 77378612 | Uncultured bacterium | **0.010** | 0.005 | 0.124 | 1.734 |
| 32895108 | Uncultured bacterium | **0.010** | 0.021 | 0.689 | 0.182 |
| 356690639 | Uncultured bacterium | **0.010** | 0.040 | 0.238 | 0.458 |
| 306488501 | Uncultured organism | **0.009** | 0.006 | 0.349 | 0.521 |
| 46981114 | *Marinobacter sp.* | **0.008** | 0.009 | 0.136 | 1.347 |
| 356690697 | Uncultured bacterium | -0.011 | 0.019 | 0.080 | 2.543 |
| 312961545 | *Pseudomonas fluorescens* | -0.012 | 0.001 | 0.150 | 2.383 |
| 74038370 | Uncultured bacterium | -0.013 | 0.018 | 0.081 | 2.670 |
| 46850218 | Uncultured bacterium | -0.030 | 0.000 | 0.285 | 2.744 |
| 255707724 | Uncultured bacterium | -1.287 | 0.046 | 0.455 | 0.238 |
| ***norB*** | | | | | |
| 376007247 | *Arthrospira sp.* | **3.018** | 0.004 | 0.992 | 0.100 |
| 157913484 | *Dinoroseobacter shibae* | **0.014** | 0.022 | 0.395 | 0.321 |
| 359345680 | *Pseudovibrio sp.* | **0.013** | 0.040 | 0.922 | 0.098 |
| 34498949 | *Chromobacterium violaceum* | **0.011** | 0.004 | 0.173 | 1.144 |
| 320150996 | *Thermus scotoductus* | **0.009** | 0.046 | 0.241 | 0.390 |
| 304568766 | *Corynebacterium accolens* | **0.008** | 0.038 | 0.099 | 1.030 |
| 339888215 | *Neisseria macacae* | **0.006** | 0.049 | 0.064 | 1.485 |
| 29466078 | Uncultured bacterium | -0.009 | 0.026 | 0.073 | 1.859 |
| 29466088 | Uncultured bacterium | -0.014 | 0.007 | 0.104 | 2.050 |
| 319742063 | *Lautropia mirabilis* | -0.014 | 0.002 | 0.130 | 2.113 |
| 29466068 | Uncultured bacterium | -0.016 | 0.004 | 0.119 | 2.112 |
| 29466006 | Uncultured bacterium | -0.018 | 0.002 | 0.140 | 2.159 |
| ***nosZ*** | | | | | |
| 322410210 | *Bacillus sp.* | **7.169** | 0.025 | 0.999 | 0.080 |
| 241765502 | *Acidovorax delafieldii* | **1.971** | 0.012 | 0.976 | 0.103 |
| 84684512 | *Rhodobacterales bacterium* | **0.065** | 0.030 | 0.732 | 0.141 |
| 261857196 | Uncultured bacterium | **0.051** | 0.000 | 0.802 | 0.269 |
| 422314453 | Uncultured bacterium | **0.039** | 0.015 | 0.115 | 1.404 |
| 422314405 | Uncultured bacterium | **0.034** | 0.003 | 0.167 | 1.327 |
| 4633570 | Uncultured bacterium | **0.031** | 0.001 | 0.693 | 0.310 |
| 373875027 | *Leptonema illini* | **0.030** | 0.025 | 0.536 | 0.237 |
| 91807154 | Uncultured α-Proteobacterium | **0.029** | 0.006 | 0.256 | 0.732 |
| 422313913 | Uncultured bacterium | **0.016** | 0.006 | 0.172 | 1.122 |
| 319945535 | *Lautropia mirabilis* | **0.016** | 0.006 | 0.125 | 1.612 |
| 374855675 | Uncultured OP1 bacterium | **0.016** | 0.001 | 0.173 | 1.645 |
| 149144841 | *Roseovarius sp.* | **0.014** | 0.011 | 0.105 | 1.571 |
| 76058199 | Uncultured bacterium | **0.013** | 0.014 | 0.972 | 0.099 |
| 399769557 | Uncultured bacterium | **0.013** | 0.025 | 0.126 | 1.026 |
| 111919356 | Uncultured bacterium | **0.012** | 0.018 | 0.153 | 0.899 |
| 422313937 | Uncultured bacterium | **0.012** | 0.017 | 0.120 | 1.195 |
| 164508588 | Uncultured bacterium | **0.012** | 0.026 | 0.077 | 1.730 |
| 393760576 | *Alcaligenes faecalis* | **0.011** | 0.044 | 0.060 | 1.962 |
| 383620376 | *Halobiforma lacisalsi* | **0.010** | 0.010 | 0.125 | 1.350 |
| 213013102 | Uncultured bacterium | **0.009** | 0.038 | 0.397 | 0.268 |
| 421953048 | Uncultured bacterium | **0.009** | 0.022 | 0.227 | 0.572 |
| 32478358 | Uncultured soil bacterium | **0.007** | 0.019 | 0.439 | 0.299 |
| 83701114 | Uncultured bacterium | **0.007** | 0.014 | 0.098 | 1.562 |
| 422314173 | Uncultured bacterium | **0.007** | 0.015 | 0.428 | 0.322 |
| 422314395 | Uncultured bacterium | **0.006** | 0.028 | 0.073 | 1.708 |
| 164508566 | Uncultured organism | -0.007 | 0.010 | 0.981 | 0.096 |
| 40362781 | Uncultured soil bacterium | -0.009 | 0.033 | 0.068 | 1.931 |
| 63081980 | Uncultured bacterium | -0.010 | 0.039 | 0.062 | 2.143 |
| 422314497 | Uncultured bacterium | -0.010 | 0.041 | 0.061 | 2.097 |
| 376372774 | Uncultured bacterium | -0.010 | 0.037 | 0.065 | 1.911 |
| 384467092 | *Citreicella sp.* | -0.011 | 0.019 | 0.080 | 2.191 |
| 409188157 | Uncultured bacterium | -0.012 | 0.020 | 0.079 | 2.033 |
| 225001297 | Uncultured bacterium | -0.012 | 0.042 | 0.060 | 2.131 |
| 226346720 | Uncultured bacterium | -0.014 | 0.045 | 0.060 | 2.111 |
| 76057545 | Uncultured bacterium | -0.015 | 0.003 | 0.121 | 2.479 |
| 409393882 | *Pseudomonas sp.* | -0.016 | 0.004 | 0.115 | 2.249 |
| 119863768 | *Psychromonas ingrahamii* | -0.016 | 0.044 | 0.060 | 2.097 |
| 226347002 | Uncultured bacterium | -0.019 | 0.000 | 0.181 | 2.302 |
| 421953192 | Uncultured bacterium | -0.020 | 0.000 | 0.220 | 2.203 |
| 4633573 | Uncultured bacterium | -0.022 | 0.000 | 0.229 | 2.345 |
| 260061020 | *Robiginitalea biformata* | -0.028 | 0.000 | 0.327 | 2.411 |
| 221635600 | *Thermomicrobium roseum* | -0.047 | 0.009 | 0.845 | 0.145 |
| 422314449 | Uncultured bacterium | -0.071 | 0.027 | 0.302 | 0.395 |
| ***napA*** | | | | | |
| 169793524 | Uncultured bacterium | **0.190** | 0.006 | 0.941 | 0.132 |
| 153864882 | *Beggiatoa sp.* | **0.130** | 0.004 | 0.658 | 0.263 |
| 157285650 | Uncultured bacterium | **0.035** | 0.007 | 0.162 | 1.216 |
| 219549420 | *Vibrio parahaemolyticus* | **0.032** | 0.015 | 0.136 | 1.208 |
| 148474786 | Uncultured bacterium | **0.030** | 0.032 | 0.330 | 0.374 |
| 169793654 | Uncultured bacterium | **0.018** | 0.019 | 0.079 | 2.226 |
| 157285630 | Uncultured bacterium | **0.015** | 0.016 | 0.198 | 0.769 |
| 257458839 | *Campylobacter gracilis* | **0.012** | 0.015 | 0.091 | 1.886 |
| 157913465 | *Dinoroseobacter shibae* | **0.006** | 0.028 | 0.078 | 1.697 |
| 148474814 | Uncultured bacterium | -0.012 | 0.030 | 0.069 | 2.232 |
| 170027493 | Uncultured bacterium | -0.015 | 0.004 | 0.118 | 2.364 |
| ***nrfA*** | | | | | |
| 157373827 | *Shewanella sediminis* | **2.055** | 0.048 | 0.666 | 0.169 |
| 114564199 | *Shewanella frigidimarina* | **0.043** | 0.033 | 0.157 | 0.856 |
| 148645204 | Uncultured Geobacter sp. | **0.035** | 0.046 | 0.185 | 0.609 |
| 154148861 | *Campylobacter hominis* | **0.033** | 0.016 | 0.720 | 0.197 |
| 197118452 | *Geobacter bemidjiensis* | **0.016** | 0.033 | 0.152 | 0.884 |
| 238712370 | *Yersinia rohdei* | **0.014** | 0.002 | 0.368 | 0.656 |
| 373849320 | *Opitutaceae bacterium* | **0.009** | 0.014 | 0.093 | 1.862 |
| 145299277 | *Aeromonas salmonicida* | **0.008** | 0.034 | 0.088 | 1.478 |
| 313896322 | *Selenomonas sp.* | **0.008** | 0.017 | 0.107 | 1.525 |
| 281300792 | *Prevotella buccalis* | **0.005** | 0.049 | 0.064 | 1.794 |
| 42524234 | *Bdellovibrio bacteriovorus* | -0.007 | 0.038 | 0.063 | 2.306 |
| 95133480 | *Desulfuromonas acetoxidans* | -0.012 | 0.009 | 0.101 | 2.353 |
| 127511439 | *Shewanella loihica* | -0.012 | 0.004 | 0.116 | 2.646 |
| 160355818 | *Bacillus selenitireducens* | -0.014 | 0.001 | 0.162 | 2.401 |
| 118579629 | *Pelobacter propionicus* | -0.017 | 0.003 | 0.122 | 2.520 |
| 258526328 | *Desulfurivibrio alkaliphilus* | -0.021 | 0.000 | 0.204 | 2.681 |
| 197088661 | *Geobacter bemidjiensis* | -0.028 | 0.042 | 0.097 | 1.319 |
| 385697272 | *Haemophilus paraphrohaemolyticus* | -0.723 | 0.013 | 0.739 | 0.187 |
| ***amoA*** | | | | | |
| 124110874 | Uncultured bacterium | **0.177** | 0.002 | 0.757 | 0.281 |
| 192822615 | Uncultured bacterium | **0.101** | 0.005 | 0.815 | 0.215 |
| 81251152 | Uncultured bacterium | **0.042** | 0.000 | 0.310 | 1.900 |
| 262206505 | Uncultured bacterium | **0.037** | 0.012 | 0.180 | 1.078 |
| 379324114 | Uncultured bacterium | **0.011** | 0.050 | 0.401 | 0.318 |
| 119654847 | Uncultured bacterium | **0.010** | 0.003 | 0.499 | 0.471 |
| 356691887 | Uncultured bacterium | **0.009** | 0.008 | 0.148 | 1.432 |
| 307748856 | Uncultured bacterium | -0.004 | 0.030 | 0.069 | 2.384 |
| 339130707 | Uncultured bacterium | -0.017 | 0.004 | 0.118 | 2.926 |
| 296920720 | Uncultured bacterium | -0.017 | 0.016 | 0.089 | 2.544 |
| 209528490 | Uncultured bacterium | -0.021 | 0.013 | 0.089 | 3.099 |
| ***hao*** | | | | | |
| 208605080 | *Nitrosomonas sp.* | **0.012** | 0.022 | 0.114 | 1.248 |
| 336453287 | *Helicobacter bizzozeronii* | -0.013 | 0.004 | 0.119 | 2.486 |
| 222354270 | Uncultured bacterium | -0.021 | 0.010 | 0.539 | 0.392 |
| 345125349 | Endosymbiont of Tevnia jerichonana | -0.022 | 0.007 | 0.111 | 2.244 |
| 91200182 | *Candidatus Kuenenia stuttgartiensis* | -0.022 | 0.012 | 0.098 | 1.897 |
| ***nifH*** | | | | | |
| 62149156 | Uncultured bacterium | **9.197** | 0.002 | 0.831 | 0.224 |
| 344327925 | *Desulfosporosinus sp.* | **3.959** | 0.002 | 0.752 | 0.264 |
| 3157632 | Unidentified nitrogen-fixing bacteria | **2.525** | 0.025 | 0.852 | 0.143 |
| 129279092 | Uncultured bacterium | **0.938** | 0.026 | 0.998 | 0.091 |
| 13173333 | Uncultured bacterium | **0.209** | 0.041 | 0.798 | 0.147 |
| 139003484 | Uncultured nitrogen-fixing bacterium | **0.139** | 0.004 | 0.466 | 0.434 |
| 110341440 | *Chlorobium ferrooxidans* | **0.108** | 0.021 | 0.327 | 0.451 |
| 139002679 | Uncultured nitrogen-fixing bacterium | **0.103** | 0.005 | 0.817 | 0.185 |
| 73534131 | Uncultured bacterium | **0.079** | 0.018 | 0.338 | 0.439 |
| 23867880 | Unidentified nitrogen-fixing bacteria | **0.071** | 0.001 | 0.776 | 0.294 |
| 78102103 | Uncultured bacterium | **0.044** | 0.007 | 0.238 | 0.807 |
| 371909423 | Uncultured bacterium | **0.041** | 0.003 | 0.217 | 1.081 |
| 3157626 | Unidentified nitrogen-fixing bacteria | **0.035** | 0.024 | 0.119 | 1.205 |
| 385258366 | Uncultured bacterium | **0.035** | 0.002 | 0.379 | 0.627 |
| 139002966 | Uncultured nitrogen-fixing bacterium | **0.028** | 0.021 | 0.109 | 1.492 |
| 37548730 | Uncultured bacterium | **0.028** | 0.007 | 0.126 | 1.701 |
| 37548698 | Uncultured bacterium | **0.025** | 0.015 | 0.097 | 1.876 |
| 78102079 | Uncultured bacterium | **0.021** | 0.008 | 0.343 | 0.520 |
| 37925797 | Uncultured bacterium | **0.019** | 0.002 | 0.186 | 1.392 |
| 62149262 | Uncultured bacterium | **0.019** | 0.039 | 0.806 | 0.131 |
| 323281987 | Uncultured bacterium | **0.017** | 0.035 | 0.127 | 0.994 |
| 3157690 | Unidentified nitrogen-fixing bacteria | **0.016** | 0.024 | 0.540 | 0.256 |
| 89474018 | Uncultured cyanobacterium | **0.015** | 0.023 | 0.603 | 0.214 |
| 44829115 | Uncultured bacterium | **0.015** | 0.023 | 0.764 | 0.167 |
| 47600976 | Uncultured bacterium | **0.014** | 0.017 | 0.130 | 1.243 |
| 37955634 | Uncultured nitrogen-fixing bacterium | **0.013** | 0.016 | 0.424 | 0.361 |
| 139003462 | Uncultured nitrogen-fixing bacterium | **0.012** | 0.000 | 0.435 | 0.782 |
| 3372194 | Unidentified bacterium | **0.012** | 0.025 | 0.753 | 0.161 |
| 99083183 | Uncultured bacterium | **0.011** | 0.043 | 0.080 | 1.484 |
| 110630580 | Uncultured soil bacterium | **0.011** | 0.035 | 0.081 | 1.611 |
| 99083393 | Uncultured bacterium | **0.011** | 0.001 | 0.187 | 1.486 |
| 290583340 | Uncultured bacterium | **0.011** | 0.006 | 0.126 | 1.683 |
| 308231247 | Uncultured bacterium | **0.009** | 0.004 | 0.191 | 1.153 |
| 60326796 | Uncultured nitrogen-fixing bacterium | **0.009** | 0.003 | 0.144 | 1.719 |
| 323365742 | Uncultured microorganism | **0.008** | 0.005 | 0.120 | 1.831 |
| 139003174 | Uncultured nitrogen-fixing bacterium | **0.007** | 0.036 | 0.094 | 1.330 |
| 66967791 | Uncultured bacterium | **0.007** | 0.036 | 0.096 | 1.278 |
| 89893108 | *Desulfitobacterium hafniense* | **0.007** | 0.047 | 0.058 | 2.155 |
| 87302506 | *Synechococcus sp.* | **0.006** | 0.028 | 0.080 | 1.774 |
| 208970502 | Uncultured bacterium | -0.006 | 0.034 | 0.065 | 2.049 |
| 372006791 | *Holophaga foetida* | -0.006 | 0.023 | 0.076 | 2.343 |
| 323365716 | Uncultured microorganism | -0.008 | 0.045 | 0.059 | 2.287 |
| 159145955 | Uncultured nitrogen-fixing bacterium | -0.009 | 0.038 | 0.063 | 2.308 |
| 139003172 | Uncultured nitrogen-fixing bacterium | -0.010 | 0.035 | 0.065 | 2.239 |
| 323365180 | Uncultured microorganism | -0.010 | 0.038 | 0.063 | 2.342 |
| 110630622 | Uncultured soil bacterium | -0.010 | 0.037 | 0.064 | 2.603 |
| 73534169 | Uncultured bacterium | -0.010 | 0.023 | 0.075 | 2.251 |
| 323366320 | Uncultured microorganism | -0.011 | 0.002 | 0.131 | 2.339 |
| 77637874 | *Alkaliphilus metalliredigenes* | -0.011 | 0.008 | 0.101 | 2.259 |
| 208970636 | Uncultured bacterium | -0.011 | 0.039 | 0.062 | 2.308 |
| 323365200 | Uncultured microorganism | -0.012 | 0.005 | 0.113 | 2.590 |
| 204304175 | Uncultured microorganism | -0.012 | 0.022 | 0.078 | 2.218 |
| 85672433 | *Roseovarius sp.* | -0.012 | 0.023 | 0.076 | 2.275 |
| 325290931 | *Syntrophobotulus glycolicus* | -0.012 | 0.014 | 0.088 | 2.363 |
| 89512880 | Uncultured nitrogen-fixing bacterium | -0.013 | 0.020 | 0.079 | 2.552 |
| 76667345 | Uncultured nitrogen-fixing bacterium | -0.013 | 0.010 | 0.096 | 2.551 |
| 6523535 | Uncultured microorganism | -0.013 | 0.045 | 0.061 | 2.283 |
| 76667453 | Uncultured nitrogen-fixing bacterium | -0.013 | 0.016 | 0.086 | 2.234 |
| 242240028 | *Dickeya dadantii* | -0.014 | 0.005 | 0.112 | 2.404 |
| 89512768 | Uncultured nitrogen-fixing bacterium | -0.014 | 0.020 | 0.080 | 2.373 |
| 89512372 | Uncultured nitrogen-fixing bacterium | -0.014 | 0.033 | 0.068 | 2.205 |
| 208343513 | Uncultured soil bacterium | -0.014 | 0.046 | 0.059 | 2.394 |
| 334128506 | *Centipeda periodontii* | -0.015 | 0.008 | 0.100 | 2.498 |
| 313895321 | *Selenomonas sp.* | -0.016 | 0.001 | 0.153 | 2.707 |
| 333737512 | *Treponema azotonutricium* | -0.017 | 0.005 | 0.113 | 2.209 |
| 3157524 | Unidentified nitrogen-fixing bacteria | -0.017 | 0.000 | 0.173 | 2.679 |
| 330839043 | S*elenomonas sputigena* | -0.017 | 0.002 | 0.129 | 2.706 |
| 323366234 | Uncultured microorganism | -0.706 | 0.044 | 0.589 | 0.186 |
| 89512876 | Uncultured nitrogen-fixing bacterium | -1.799 | 0.018 | 0.999 | 0.080 |
