# Supplementary material for: Microbial Functional Gene Diversity Predicts Groundwater Contamination and Ecosystem Functioning
Source: mBio. 2018 Feb 20;9(1):e02435-17. doi: 10.1128/mBio.02435-17 (PMC5821090; doi:10.1128/mBio.02435-17)
Supplement: TABLE S5 [file mbo001183730st5.docx]

**Table S5** Automatically selected 50 predictors from 2,361 detected functional genes related to uranium reduction by AUS-RF [^31^](#_ENREF_31) for predicting uranium contamination in groundwater. Bold items were also identified as significantly increased/decreased populations with increasing uranium concentrations in groundwater (Table S3).

| **GenBank ID** | **Importance** | **Functional category** | **Gene/enzyme** | **Derived organism** |
| --- | --- | --- | --- | --- |
| 88643378 | 0.1858 | Sulfite reduction | *dsrB* | Uncultured prokaryote |
| 219965663 | 0.1710 | Sulfite reduction | *dsrA* | Uncultured sulfate-reducing bacterium |
| 334338986 | 0.1673 | Sulfite reduction | *dsrB* | *Desulfotomaculum ruminis* |
| 118582234 | 0.1407 | Electron transfer | Hydrogenase | *Shewanella decolorationis* |
| 20502031 | 0.1403 | Sulfite reduction | *dsrA* | Uncultured sulfate-reducing bacterium |
| 144905819 | 0.1387 | Sulfite reduction | *dsrA* | Uncultured sulfate-reducing bacterium |
| 76666836 | 0.1346 | Sulfite reduction | *dsrB* | Uncultured sulfate-reducing bacterium |
| 37726837 | 0.1338 | Sulfite reduction | *dsrA* | Uncultured prokaryote |
| 119774344 | 0.1332 | Electron transfer | Cytochrome | *Shewanella amazonensis* |
| 157318765 | 0.1166 | Electron transfer | Cytochrome | *Shewanella sediminis* |
| 303282825 | 0.1153 | Sulfite reduction | *sir* | *Micromonas pusilla* |
| 46307848 | 0.1147 | Sulfite reduction | *dsrA* | Uncultured sulfate-reducing bacterium |
| 13249519 | 0.1124 | Sulfite reduction | *dsrB* | Uncultured sulfate-reducing bacterium |
| 34017154 | 0.1045 | Sulfite reduction | *dsrA* | Uncultured bacterium |
| 219939239 | 0.1024 | Sulfite reduction | *dsrA* | Uncultured sulfate-reducing bacterium |
| 127511504 | 0.1013 | Electron transfer | Cytochrome | *Shewanella loihica* |
| 109452398 | 0.1008 | Sulfite reduction | *dsrA* | Uncultured sulfate-reducing bacterium m |
| 224612976 | 0.0990 | Sulfite reduction | *dsrB* | *Thermodesulfatator atlanticus* |
| 63029785 | 0.0939 | Sulfite reduction | *dsrB* | Uncultured sulfate-reducing bacterium |
| 118424334 | 0.0892 | Sulfite reduction | *dsrA* | Uncultured sulfate-reducing bacterium |
| 259021639 | 0.0884 | Electron transfer | Hydrogenase | *Rhodobacter sp.* |
| 84778345 | 0.0872 | Sulfite reduction | *dsrA* | Uncultured sulfate-reducing bacterium |
| 225194089 | 0.0863 | Sulfite reduction | *dsrB* | Uncultured sulfate-reducing bacterium |
| **218885637** | **0.0856** | **Electron transfer** | **Cytochrome** | ***Desulfovibrio vulgaris*** |
| 307742753 | 0.0853 | Sulfite reduction | *dsrB* | Uncultured sulfate-reducing bacterium |
| 153007810 | 0.0842 | Electron transfer | Cytochrome | *Ochrobactrum anthropi* |
| 46519969 | 0.0824 | Sulfite reduction | *dsrA* | Uncultured sulfate-reducing bacterium |
| 393165628 | 0.0820 | Electron transfer | Cytochrome | *Alcaligenes faecalis* subsp. faecalis |
| 379989495 | 0.0812 | Sulfite reduction | *dsrB* | Uncultured bacterium |
| 189424294 | 0.0803 | Electron transfer | Cytochrome | *Geobacter lovleyi* |
| 116070689 | 0.0801 | Sulfite reduction | *sir* | *Synechococcus sp.* |
| 109452399 | 0.0797 | Sulfite reduction | *dsrB* | Uncultured sulfate-reducing bacterium |
| 46307932 | 0.0787 | Sulfite reduction | *dsrA* | Uncultured sulfate-reducing bacterium |
| **46307852** | **0.0780** | **Sulfite reduction** | ***dsrA*** | **Uncultured sulfate-reducing bacterium** |
| 78357899 | 0.0772 | Electron transfer | Cytochrome | *Desulfovibrio desulfuricans* |
| 191161439 | 0.0764 | Electron transfer | Cytochrome | *Geobacter sp.* |
| 15077478 | 0.0762 | Sulfite reduction | *dsrB* | *Desulfofaba gelida* |
| **157679062** | **0.0761** | **Sulfite reduction** | ***dsrA*** | **Uncultured sulfate-reducing bacterium** |
| **46307906** | **0.0760** | **Sulfite reduction** | ***dsrA*** | **Uncultured sulfate-reducing bacterium** |
| 116743209 | 0.0738 | Sulfite reduction | *dsrB* | Uncultured sulfate-reducing bacterium |
| 225194053 | 0.0707 | Sulfite reduction | *dsrB* | Uncultured sulfate-reducing bacterium |
| 308750092 | 0.0704 | Electron transfer | Cytochrome | *Enterobacter cloacae* |
| 300332686 | 0.0694 | Sulfite reduction | *sir* | *Oscillatoria sp.* |
| 83312466 | 0.0671 | Sulfite reduction | *dsrB* | *Magnetospirillum magneticum* |
| 229362307 | 0.0670 | Electron transfer | Cytochrome | *Pseudomonas fluorescens* |
| 313587715 | 0.0660 | Sulfite reduction | *dsrB* | Uncultured sulfate-reducing bacterium |
| 379988587 | 0.0657 | Sulfite reduction | *dsrB* | Uncultured bacterium |
| 197085806 | 0.0632 | Electron transfer | Cytochrome | *Geobacter bemidjiensis* |
| 222056817 | 0.0630 | Electron transfer | Cytochrome | *Geobacter sp.* |
| 2329993 | 0.0627 | Sulfite reduction | *dsrA* | *Desulfovibrio simplex* |
