# Supplementary material for: Microbial Functional Gene Diversity Predicts Groundwater Contamination and Ecosystem Functioning
Source: mBio. 2018 Feb 20;9(1):e02435-17. doi: 10.1128/mBio.02435-17 (PMC5821090; doi:10.1128/mBio.02435-17)
Supplement: TABLE S6 [file mbo001183730st6.docx]

**Table S6** Automatically selected 54 predictors from 5,273 detected N cycling genes by AUC-RF [^31^](#_ENREF_31) for predicting nitrate contamination in groundwater ordered. Bold items were also identified as significantly increased/decreased populations with increasing nitrate concentrations in groundwater (Table S4).

| GenBank ID | Importance | Function category | Gene | Affiliated organism |
| --- | --- | --- | --- | --- |
| 117188459 | 0.0885 | Assimilatory N reduction | *nasA* | Uncultured eukaryote |
| **242240028** | **0.0883** | **Nitrogen fixation** | ***nifH*** | ***Dickeya dadantii*** |
| 116013350 | 0.0823 | Denitrification | *nirS* | Uncultured bacterium |
| 254786902 | 0.0804 | Assimilatory N reduction | *nirB* | *Teredinibacter turnerae* |
| 347581769 | 0.0783 | Nitrification | *amoA* | NA* |
| 194596437 | 0.0750 | Nitrification | *amoA* | NA |
| 383620376 | 0.0719 | Denitrification | *nosZ* | *Halobiforma lacisalsi* |
| 219864290 | 0.0715 | Ammonification | *ureC* | *Cyanothece sp.* |
| 296920716 | 0.0707 | Nitrification | *amoA* | NA |
| **109457400** | **0.0625** | **Assimilatory N reduction** | ***nasA*** | ***Roseobacter denitrificans*** |
| 76058434 | 0.0621 | Denitrification | *nosZ* | Uncultured bacterium |
| 213013134 | 0.0587 | Denitrification | *nosZ* | Uncultured bacterium |
| 4633553 | 0.0577 | Denitrification | *nosZ* | Uncultured bacterium |
| 71402610 | 0.0577 | Assimilatory N reduction | *nasA* | *Lyngbya aestuarii* |
| **192764352** | **0.0571** | **Denitrification** | ***narG*** | **Uncultured bacterium** |
| **60326796** | **0.0570** | **Nitrogen fixation** | ***nifH*** | **Uncultured N-fixing bacterium** |
| 52306400 | 0.0567 | Denitrification | *norB* | *Mannheimia succiniciproducens* |
| 45386157 | 0.0563 | Denitrification | *narG* | Unidentified bacterium |
| **296920720** | **0.0559** | **Nitrification** | ***amoA*** | **NA** |
| 163783644 | 0.0544 | Denitrification | *nosZ* | *Hydrogenivirga sp.* |
| 306487857 | 0.0531 | Denitrification | *nirS* | Uncultured organism |
| 359406214 | 0.0521 | Ammonification | *gdh* | *Prevotella stercorea* |
| 139003600 | 0.0513 | Nitrogen fixation | *nifH* | Uncultured N-fixing bacterium |
| 60326776 | 0.0510 | Nitrogen fixation | *nifH* | Uncultured N-fixing bacterium |
| 148254273 | 0.0503 | Assimilatory N reduction | *nasA* | *Bradyrhizobium sp.* |
| 372985287 | 0.0490 | Ammonification | *ureC* | *Oceanimonas sp.* |
| 284390204 | 0.0490 | Denitrification | *narG* | Uncultured bacterium |
| 209401906 | 0.0488 | Denitrification | *narG* | Uncultured bacterium |
| 78093560 | 0.0477 | Denitrification | *narG* | Uncultured bacterium |
| 37925891 | 0.0474 | Nitrogen fixation | *nifH* | Uncultured bacterium |
| 45386147 | 0.0472 | Denitrification | *narG* | Uncultured bacterium |
| 87300726 | 0.0472 | Assimilatory N reduction | *nasA* | *Synechococcus sp.* |
| 73762919 | 0.0468 | Denitrification | *nirK* | Uncultured bacterium |
| 20977654 | 0.0462 | Nitrogen fixation | *nifH* | Uncultured N-fixing bacterium |
| 22293726 | 0.0453 | Ammonification | *ureC* | *Thermosynechococcus elongatus* |
| 139003429 | 0.0452 | Nitrogen fixation | *nifH* | Uncultured N-fixing bacterium |
| 357387348 | 0.0449 | Ammonification | *gdh* | *Kitasatospora setae* |
| 359799482 | 0.0446 | Denitrification | *nosZ* | *Achromobacter arsenitoxydans* |
| 158306363 | 0.0438 | Assimilatory N reduction | *nirA* | *Acaryochloris marina* |
| **116634769** | **0.0429** | **Denitrification** | ***narG*** | **Uncultured bacterium** |
| 88779207 | 0.0428 | Ammonification | *ureC* | *Reinekea sp.* |
| 3805970 | 0.0425 | Dissimilatory N reduction | *napA* | *Rhodopseudomonas sp.* |
| 129279082 | 0.0419 | Nitrogen fixation | *nifH* | Uncultured bacterium |
| 32188205 | 0.0416 | Assimilatory N reduction | *nasA* | *Sphingomonas elodea* |
| 73763046 | 0.0415 | Denitrification | *nirK* | Uncultured bacterium |
| 37955640 | 0.0414 | Nitrogen fixation | *nifH* | Uncultured N-fixing bacterium |
| 118579629 | 0.0412 | Dissimilatory N reduction | *nrfA* | *Pelobacter propionicus* |
| 256790451 | 0.0411 | Dissimilatory N reduction | *nrfA* | *Slackia heliotrinireducens* |
| 91200182 | 0.0407 | Nitrification | *hao* | *Candidatus Kuenenia stuttgartiensis* |
| 71383714 | 0.0407 | Assimilatory N reduction | *nasA* | Symploca atlantica |
| 375141882 | 0.0407 | Ammonification | *ureC* | *Mycobacterium rhodesiae* |
| 138894322 | 0.0406 | Denitrification | *nirK* | *Geobacillus thermodenitrificans* |
| 157401497 | 0.0404 | Nitrogen fixation | *nifH* | *Methanococcus maripaludis* |
| 151280243 | 0.0401 | Ammonification | *ureC* | *Janthinobacterium sp. Marseille* |

NA: unidentified organism.
